# Immune Conversion of Tumor Microenvironment by Oncolytic Viruses: The Protoparvovirus H-1PV Case Study

**DOI:** 10.3389/fimmu.2019.01848

**Published:** 2019-08-07

**Authors:** Antonio Marchini, Laurent Daeffler, Vitaly I. Pozdeev, Assia Angelova, Jean Rommelaere

**Affiliations:** ^1^Laboratory of Oncolytic Virus Immuno-Therapeutics, Luxembourg Institute of Health, Luxembourg, Luxembourg; ^2^Laboratory of Oncolytic Virus Immuno-Therapeutics, German Cancer Research Center, Heidelberg, Germany; ^3^Université de Strasbourg, IPHC, Strasbourg, France; ^4^CNRS, UMR7178, Strasbourg, France; ^5^Infection, Inflammation and Cancer Program, German Cancer Research Center, Heidelberg, Germany

**Keywords:** oncolytic viruses, H-1PV, immunotherapy, immunogenic cell death, combination therapy, tumor microenvironment, checkpoint blockade

## Abstract

Cancer cells utilize multiple mechanisms to evade and suppress anticancer immune responses creating a “cold” immunosuppressive tumor microenvironment. Oncolytic virotherapy is emerging as a promising approach to revert tumor immunosuppression and enhance the efficacy of other forms of immunotherapy. Growing evidence indicates that oncolytic viruses (OVs) act in a multimodal fashion, inducing immunogenic cell death and thereby eliciting robust anticancer immune responses. In this review, we summarize information about OV-mediated immune conversion of the tumor microenvironment. As a case study we focus on the rodent protoparvovirus H-1PV and its dual role as an oncolytic and immune modulatory agent. Potential strategies to improve H-1PV anticancer efficacy are also discussed.

## Introduction

After the market approval of Imlygic® (Talimogene laherparepvec, T-Vec, Amgen, Thousand Oaks, CA, USA) (1), oncolytic viruses (OVs) are gaining tangible momentum as a new class of anticancer agents. This is apparent from the fact that more than 40 OVs belonging to at least ten viral families are currently undergoing clinical trials against various malignancies, as monotherapy or in combination with other anticancer modalities (2). Most likely, other OVs will soon be approved for use as novel therapeutics for cancer patients.

OVs selectively replicate in and kill tumor cells in a multimodal fashion while sparing normal tissues. Productive virus infection ends with the lysis of the cancer cell and the release of progeny viral particles. In this way, OVs have the ability to multiply and spread throughout the tumor bed. Importantly, OV-mediated cell death is often immunogenic and accompanied by the activation of anticancer immune responses (3). The relevance of this immunological facet of oncolytic virotherapy is further emphasized by the limited OV propagation observed in cancer patients (4).

In this review we provide a brief introduction of the tumor microenvironment, its immune components and the different strategies developed by tumors to avoid attack from the immune system, before focusing on the ability of OVs to act as immune adjuvants and contribute to the induction of systemic antitumor immunity. We also discuss possible ways to enhance the anticancer activity of OVs by combining them with other anticancer treatments and in particular with other forms of immunotherapy (e.g., checkpoint blockade). We use the protoparvovirus H-1PV, one of the OVs presently under evaluation in the clinic, as a case study.

## The Tumor Microenvironment

Solid malignant tumors comprise not only a heterogeneous population of neoplastic cells but also a multitude of resident and infiltrating non-transformed cells, secreted factors and extracellular matrix (ECM) proteins, which altogether constitute the tumor microenvironment (TME) (5). The non-transformed cells of the TME consist in particular of cancer-associated fibroblasts (CAFs), adipocytes, stromal, and vascular endothelial cells, pericytes, lymphatic endothelial cells, and recruited cells of the immune system. Tumor-infiltrating immune cells include T-lymphocytes [CD8^+^ cytotoxic (memory) T-cells, CD4^+^ helper (Th1, Th2) T-cells, and regulatory T-cells (Tregs)], B lymphocytes (B-cells), tumor-associated macrophages (TAMs), tumor-associated neutrophils (TANs), myeloid-derived suppressor cells (MDSCs), dendritic cells (DCs), and natural killer (NK) cells (5, 6).

Non-neoplastic cells may account for more than 50% of the total tumor mass, and their composition varies between different tumors. Like cancer cells, non-malignant cells produce, and release cytokines, chemokines, growth factors, matrix remodeling enzymes, vesicles, and other soluble factors into the tumor mass, often supporting tumor growth (5). Metabolic interactions between cancer and non-malignant cells influence all stages of carcinogenesis.

The ECM network, an important TME component, consists of a flexible deposit of collagen and fibronectin fibrils associated with glycoproteins, proteoglycans, and polysaccharides within and around tumor areas (7). The ECM not only serves as a physical scaffold for all cells of the TME, but also provides biochemical signals by hosting growth factors and chemokines modulating tumor cell growth, migration, and metastasis (7, 8). Although the formation of the ECM is primarily the responsibility of CAFs, cancer cells also contribute. Cancer development and progression are associated with increased ECM deposition (7).

Recent findings from whole-genome sequencing and microRNA expression profiling studies (9, 10) have further highlighted the key role of non-malignant cells and other TME components in influencing tumor growth, immune tolerance, metastasis, and therapeutic resistance (11, 12). It follows that targeting these “normal” elements may represent a new approach to complement conventional therapies and develop innovative and more efficient treatments against cancer.

## Anti-Tumor Immune Response

Among the non-transformed cells of the TME, immune cells have attracted the most attention in the past decade and have become the subject of intense preclinical and clinical research. In a healthy body, the immune system is able to detect and eliminate malignant cells (13), a phenomenon referred to as immune surveillance against tumors. The two main components of this surveillance are activated cytotoxic CD8^+^ T cells (13, 14) (also called cytotoxic T lymphocytes, CTLs) and NK cells (15) which belong, respectively, to the adaptive and innate arms of the immune system.

- In order to exert their tumoricidal activity, CTLs have to recognize tumor-associated antigen (TAAs) motifs presented by major histocompatibility complex class I molecules (MHC-I) on tumor cells. To become activated, naïve CTL need to be previously primed by professional, antigen-presenting cells (APCs) which expose TAA motifs through MHC-I molecules to T-cell receptors on CTLs. CD28 molecules expressed at the surface of CTLs bind to CD80 or CD86 polypeptides exposed on APCs (DCs or macrophages), providing a co-stimulatory signal for CTL killing activation. CTL tumoricidal activity is carried out both directly through the release of cytotoxic granules containing perforin and granzymes, and indirectly through the secretion of cytokines such as interferon-γ (IFN-γ), tumor necrosis factor-α (TNF-α), and IL-2. These cytokines induce apoptosis of tumor cells and/or activation of anticancer immune responses (16). CD4^+^ T helper cells also contribute to the cytotoxic anticancer immune response mediated by CTLs, by stimulating CTL priming through the release of cytokines, particularly IFN-γ (17).
- In contrast to CTLs, NK cells do not require specific TAA recognition to interact with tumor cells nor MHC-dependent cross-priming. A repertoire of inhibitory and activating receptors on these cells, makes their activity dependent on the down- or up-modulation of various ligands exposed on tumor cells, respectively. Similarly to CTL, NK cells are able to kill neoplastic cells directly by releasing perforin and granzymes as well as indirectly by secreting death receptor ligands (FasL and TRAIL) and cytokines [IFN-γ, TNF-α, and granulocyte macrophage-colony-stimulating factor (GM-CSF)] (15). NK cell functions can be activated or exacerbated in presence of cytokines released by DCs and monocytes (IL-12 and IL-15) as well as T-cells or NK cells themselves (IL-2) (18).

In conclusion, the immune system appears to play a major pleiotropic role in the surveillance against tumors.

## Tumor Strategies of Immune Evasion

As mentioned above, CD8^+^ T-cells, NK cells, and monocytes populate TMEs. The presence of these cells was found to correlate with a better prognosis and treatment responsiveness of various tumors including brain, hepatocellular, lung, breast, renal, colorectal cancers, and melanoma (19, 20). TMEs containing these immune cell populations are called inflamed. Unfortunately, the immunosuppressive ecosystem prevailing in many TMEs suppresses NK and CTL cytotoxic activities, thereby precluding long-standing protective immunity. In addition, the TME often inhibits T-cell proliferation, promotes T-cell apoptosis, down-regulates expression of MHC molecules and antigen processing machinery components on most cells within tumors (in particular neoplastic cells, DCs, and CD4^+^ T helper cells) and corrupts TAMs toward an M2 immunosuppressive phenotype, thereby allowing tumor cells to escape attack from the immune system. For a comprehensive discussion of the strategies developed by cancer cells to escape immune surveillance, we redirect readers to excellent recent reviews by Muhn and Bronte (21) and Fearon (22). Briefly, a main mechanism by which tumors prevent attack from the immune system consists in the release within TMEs of immunosuppressive molecules such as growth factors [e.g., transforming growth factor (TGF)-β], cytokines [e.g., interleukin-10 (IL-10)], chemokines, inflammatory, and matrix-remodeling enzymes as well as metabolites. These molecules contribute to establish complex and dynamic communication networks between all the cells composing a tumor in order to promote its survival, development, and metastasis. These molecules are produced not only by tumor cells, but also by non-malignant cells of the TME including CAFs (23, 24), adipocytes (25), and infiltrating immune cells such as Tregs (26, 27), Bregs (28, 29), MDSCs (30, 31), and TAMs (32). Thus, diverse cell subtypes depending on their activation state by producing and secreting these molecules simultaneously participate in establishing an immune-suppressive TME *via* multiple mechanisms [e.g., Tregs through the production of IL-10 and TGF-β inhibit CTL and NK cytolytic activity, promote Treg survival, and expansion and modulate the activity of other immunosuppressive cells within the TME such as Bregs, MDSCs, TAMs and CAF, which in their turn concur to augment immunosuppression (26, 27)]. The activity of these cells may change from tumor to tumor and during the different phases of tumorigenesis and even between different regions within the same tumor.

A second immune-inhibitory mechanism relies on a natural process developed by the immune system to regulate the amplitude and the quality of the T-cell response. This mechanism is triggered to prevent the immune response from getting over-activated and causing autoimmune reactions that could damage healthy tissues. The factors involved in this inhibitory process are collectively referred to as immune checkpoint molecules (ICs) and are expressed at the surface of several cell populations of TME. The mechanism is triggered upon interaction of ICs acting as receptors and located on tumor-infiltrating effector T-cells, B-cells and NK cells, with specific ICs behaving as ligands and often expressed at the surface of APCs, Tregs, TAMs, and MDSCs. Interestingly, ICs ligands are overexpressed in many tumor cells. Well-known examples of IC-receptors include the CTL-associated antigen 4 (CTLA-4/CD152), the programmed death receptor 1 (PD-1/CD279), and the molecules lymphocyte activation gene-3 (LAG-3), T cell immunoglobulin, and mucin domain containing protein 3 (TIM-3), and T-cell immunoreceptor with Ig and ITIM domains (TIGIT) (33–35). The corresponding ligands are CD80 and CD86 for CTLA-4, and programmed death receptor ligand 1 and 2 (PD-L1/CD274, PD-L2/CD273) for PD-1. These IC receptor-ligand interactions play a critical role in blocking anticancer immune responses mediated by cytotoxic T-cells and NK cells in TMEs (35). The underlying molecular mechanisms involved in these inhibitory signaling pathways are complex and beyond the scope of this review (36–38). Within the TME, tumor cells and myeloid cells are considered to be the main cell types responsible for T-cell suppression through the expression of PD-1 ligands (39).

## Cancer Immunotherapy

To overcome tumor-driven immune evasion and suppression, a new appealing therapeutic strategy, namely cancer immunotherapy, emerged, and was recognized as the breakthrough of the year 2013 (40). Presently, the field is rapidly expanding, yielding continuously growing evidence of clinical efficiency in patients with various types of solid and hematological tumors. Cancer immunotherapy is generally based on two approaches. Passive immunotherapy aims at enhancing an already existing antitumor immune response; active immunotherapy attempts to trigger the latter *de novo*. Administration of immunomodulating antibodies (e.g., immune checkpoint inhibitors, ICIs) and the adoptive transfer of tumor-infiltrating lymphocytes or chimeric antigen receptor (CAR) T-cells represent the passive immunotherapy approach, while the active one is exemplified by anticancer vaccination [discussed in this issue by Fennemann et al. (41)]. Current cancer immunotherapeutic strategies, their molecular bases, challenges, and future directions and prospects are extensively reviewed in various recent publications to which the reader is redirected (41–44). Special attention is paid in the present review to immune checkpoint blockade using ICIs, given its relevance to the oncolytic virotherapy approaches discussed below.

## Immune Checkpoint Blockade

The discovery of the aforementioned immunosuppressive pathways represented a breakthrough for oncology, as illustrated by the 2018 Nobel Prize in Physiology or Medicine jointly awarded to Allison and Honjo for their contribution to novel cancer therapy approaches based on the inhibition of negative immune regulation. Indeed, these findings have paved the way for the development of innovative treatments that aim to restore or boost anticancer immune responses in TMEs through alleviation of the immunosuppressive signals inhibiting the cytotoxic activities of CTL and NK cells (35). Like other promising cancer immunotherapies using DC-based vaccines (45) and CAR T-cell therapy (46), the application of immune checkpoint inhibitors is currently the subject of intense efforts worldwide to harness the power of the immune system against cancers.

While small-molecule immune checkpoint inhibitors are under development (47), immune checkpoint blockade (ICB) has been successfully achieved using monoclonal antibodies that interfere with the interactions between checkpoint receptors and cognate ligands by targeting either of these molecules (48). Examples of ICB include nivolumab and pembrolizumab directed against PD-1; ipilimumab specific for CTLA-4; and atezolizumab, durvalumab, and avelumab developed against PD-L1. These market-approved antibodies, alone or in combination, showed impressive results against several types of cancer including melanoma and lung carcinomas (48, 49), with some patients experiencing a durable and complete anticancer response. New antibodies targeting the more recently discovered immune checkpoint molecules Tim-3 (50) and LAG-3 (33) have shown pre-clinical efficacy and are now entering clinical trials (51, 52).

Despite these successes, it should be stated that only a fraction (10–40%) of treated patients responds positively to checkpoint blockade with PD-1 or PD-L1 specific antibodies (53). In addition treatment resistance is common (54, 55) influenced at least in part by patient HLA class I genotype (56). Furthermore, the appearance of severe immune-related adverse events due to an exacerbated activation of the global immune system (57, 58) hampers (combinatorial) treatments with checkpoint blocking antibodies.

The clinical outcome of checkpoint blockade is thought to depend on the neoantigen load of tumors as well as the size and composition of the immune cell population present in the tumor bed. Inflamed tumors (also referred to as hot tumors) that contain CD8^+^ and CD4^+^ T-cells, monocytes and pro-inflammatory cytokines, show the best response rate to ICB (59). Indeed, the immune landscape of inflamed tumors is indicative of a pre-existing antitumor immune response that has been silenced by the tumor-bed suppressive environment, as revealed by prominent Treg and MDSC infiltration, production of anti-inflammatory cytokines or T cell exhaustion. Another common feature of inflamed tumors is the elevated expression of PD-L1 by neoplastic or immune cells. A PD-1/PD-L1 signature in tumors generally correlates with a positive response to anti-PD-1 therapy (19), although PD-L1 expression is not a prerequisite for successful checkpoint therapy.

In contrast, immune-excluded or deserted tumors (cold tumors) are characterized by poor or almost no T-cell infiltration in the stroma, and they respond poorly to ICB (20, 60). Therefore, it is clear that the development of new strategies to convert a cold TME into a hot one, is essential for improving the clinical outcome of ICB and increasing the proportion of patients who benefit from this treatments. One of the most promising strategies in this respect is the use of OVs.

## Oncolytic Viruses

In recent years, OVs have attracted significant attention as anti-cancer agents because they preferably replicate in, and eventually lyse, tumor cells while sparing normal counterparts. Tumor cells offer a favorable environment for the lytic replication of many OVs that exploit various physiological alterations occurring in cancer cells. These tumor cell defects are often associated with: (i) rapid proliferation and dysregulated metabolism (61); (ii) impairment of antiviral immune responses (62); (iii) production of immune suppressive factors in the TME (63, 64), (iv) intracellular signaling pathway alterations that promote survival under stress conditions (65, 66). Besides directly killing tumor cells through activation of different cytocidal programs ranging from apoptosis, pyroptosis, and necroptosis to autophagy and lysosome-dependent cell death, OVs proved able to convert a cold TME into an inflamed one, thereby reawakening antitumor immune responses. Due to their multimodal activity, OVs have become a major focus of interest in cancer therapy research. As a result of their oncosuppressive activities, more than forty OVs are presently in clinical testing against various malignancies and a number of OVs are undergoing phase III clinical trials (67).

This list of OVs under investigation includes herpes simplex virus (HSV), adenoviruses (Ad), vaccinia virus (VV), measles virus (MV), coxsackie virus, poliovirus, protoparvovirus, reovirus, Newcastle disease virus, vesicular stomatitis virus (VSV), and Seneca Valley virus. Some of the OVs undergoing clinical trials are based on human pathogens (e.g., Ad, HSV, MV, poliovirus) and are engineered to reduce their toxicity and compel their lytic multiplication in response to factors and/or pathways specifically active in tumor cells.

The therapeutic potential of OVs can be best exemplified by the clinical benefit of the prototypical drug in this class, the genetically modified type 1 HSV designated talimogene laherparepvec (T-Vec). For a recent T-Vec review, the reader is referred to Conry et al. (68). Based on encouraging clinical results, T-Vec became the first oncolytic virus to receive regulatory approval by FDA in 2015 with an indication for advanced melanoma (1). This virus was engineered to prevent production of both its neurovirulence protein ICP34.5 required for lytic infection of normal cells (in particular neurons), and its ICP47 protein that reduces MHC class I expression and virus/tumor antigen presentation by infected cells. These changes also brought the viral US11 gene under control of an early/intermediate promoter, partially reinvigorating the virus lytic activity in tumor cells. Furthermore, two copies of the human granulocyte-macrophage colony-stimulating factor (GM-CSF) gene were introduced into the virus genome to enhance it immunogenicity, knowing that GM-CSF production by cancer cells attracts DCs in the tumor niche and enhances their antigen presentation function. T-Vec propagates preferentially in neoplastic cells in which the malignant transformation process has impaired the PKR (e.g., through oncogenic Ras activation) and/or type I IFN pathways, features characterizing in particular many melanoma cells.

In a phase III randomized clinical trial (OPTiM) conducted in 436 patients with stage IIIB to IV melanoma, intralesional injection of T-Vec resulted in significantly better durable and overall response rates compared with subcutaneous administration of GM-CSF alone, with superior overall survival for patients with stage III or IV M1a disease. The virus treatment proved also to be beneficial against non-injected lesions, demonstrating its ability to stimulate an anticancer immune response. In T-Vec-treated patients with cutaneous melanoma arising in the head and neck, a complete response rate of 30% was achieved, and 73% of responses persisted longer than 1 year. Based on these results, phase IB/II and IB/III clinical trials combining T-Vec with the immune checkpoint inhibitors ipilimumab (anti-CTLA-4) or pembrolizumab (anti-PD-1), respectively, were undertaken in patients with advanced melanoma (see below section Strategies to improve OVs and optimize their immune stimulatory activities).

Another promising candidate for clinical applications is the oncolytic poliovirus PVSRIPO in which the internal ribosomal entry site of poliovirus is replaced with that of human rhinovirus type 2, to ablate neurovirulence. PVSRIPO uses for its entry the poliovirus receptor CD155, which is highly expressed on the surface of neoplastic cells and in other cells of the TME. There is preclinical and clinical evidence that this OV has strong ability to activate DCs and promote formation of tumor-specific CTLs (69). The results of a clinical trial in which 61 patients with grade IV malignant glioma were treated with PVSRIPO, showed increased survival rate (at 24 and 36 months) in 21% of patients in comparison with historical controls (70).

Other OVs are endowed with an intrinsic oncotropism, which can be traced back to their elevated sensitivity to the antiviral innate immune responses developed by normal human cells but often deficient in their neoplastic derivatives (e.g., VSV) and/or to the depending of their lytic multiplication on oncogenic pathways [e.g., activated Ras signaling for reovirus (66)]. The group of genuinely oncotropic OVs also includes non-human animal viruses (e.g., Newcastle disease virus or protoparvovirus whose natural hosts are avian or rodent species, respectively). One advantage of animal OVs lies in the lack of pre-existing antiviral immunity in contrast to human pathogen-based OVs against which patients may have developed neutralizing antibodies prior to virotherapy. For a complete list of OVs undergoing clinical testing we redirect the reader to these recent reviews (4, 71).

It is worth noting that no champion has emerged yet among the various OVs under investigation. Each OV has indeed its own peculiar modes of replication, action, and tumor specificity. This variation justifies the continued development and optimization of these ground-breaking anticancer agents.

## Oncolytic Viruses as Tools to Heat Up Tumors

OVs evoke anticancer immune responses through different mechanisms. In addition to releasing progeny virions into the TME, virus-mediated tumor cell lysis disseminate a wide repertoire of both cellular tumor-associated antigens/neo-antigens (TAAs/TANs), danger-associated molecular patterns (DAMPs) and viral pathogen-associated molecular patterns (PAMPs) which lead to an inflammatory immune response. In an ideal scenario, TAAs and TANs are captured and processed by infiltrating APCs, in particular DCs. DCs loaded with antigens migrate to draining lymph nodes where they mature and acquire the capacity to prime T-cells, thus leading to a cancer-specific T-cell response potentially directed against a wide spectrum of tumor antigens.

PAMPs consist of viral RNA, DNA, or proteins that are sensed by pattern recognition receptors (PRRs) expressed by DCs. PRRs include Toll-like receptors, RIG-like receptors, NOD-like receptors, and cGAS (72–74). As a consequence of PRR engagement, DCs produce pro-inflammatory (e.g., TNF-α and IL-12) and antiviral [type I IFNs (IFN-α and IFN-β)] cytokines (75). These cytokines contribute to TAA/TAN cross-presentation and priming of CTL, among other effects (76). It is noteworthy that the cGAS-Sting pathway in tumor-infiltrating DCs can also sense tumor-derived genomic DNA, leading to IFN-β production and eventually CTL activation (77), highlighting the relevance of this antiviral pathway in cancer development and therapy (78).

Interestingly, OV-infected cancer cells may sense PAMPs and contribute in a direct way to the production and release of pro-inflammatory cytokines into the TMEs. This is exemplified by the type I IFN response. Neoplastic transformation is often associated with defects in antiviral innate immunity, with cancer cells unable to produce type I IFN and/or to respond to these cytokines. However, in the context of heterogeneous tumors, a fraction of cancer cells may still be able to detect viral PAMPs through their PRRs and sustain significant type I IFN production. The virus-induced type I IFN response is pleiotropic and comprises facets which are undesirable (antiviral effects) and desirable (anticancer effects) in the context of oncolytic virotherapy. Type I IFNs thus act as a double-edged sword: on the one hand, they are directed against the virus by blocking its multiplication and inducing its neutralization and elimination and on the other hand, they have anticancer properties. The oncosuppressive potential of type I IFNs relies in part on their ability to arrest tumor cell proliferation and exert anti-angiogenic effects (79–81). Furthermore, type I IFNs may promote the activation of anti-tumor immune reactions (76). It is well-documented that type I IFNs are important regulators of NK cell and CTL functions. In particular, type I IFNs stimulate NK cell cytotoxic activity and NK cell-mediated production and secretion of IFN-γ (82, 83). Besides inhibiting angiogenesis and inducing cell cycle arrest and apoptosis of tumor cells (84), IFN-γ is a strong immune stimulant. In particular, INF-γ induces the expression of MHC class II molecules on DCs, activates and increases the phagocytic activity of macrophages, and promotes antigen-specific Th1 and CTL responses (84). Type I IFNs also have a crucial role in mediating the interplay between innate and adaptive immunity. Type I IFNs induce maturation of DCs, in particular upregulating the surface expression of MHC class I and co-stimulatory CD40 and CD86 molecules, which are essential for CTL activation (85). Type I IFNs support CTL differentiation and expansion (86, 87). Accordingly, virus-induced type I IFNs directly stimulate the cross-priming of CTLs by DCs (88), and are essential for the protection of activated T cells from NK cell cytotoxicity (89). Notably, IFN-α enhances the induction and maintenance of a Th1 response through its direct action on Th-cells (90).

OVs differ markedly in the ability to trigger production of type I IFNs. The type I IFN response is, for instance a main determinant of whether melanoma cells are resistant or sensitive to oncolytic MV (91). Whereas, NDV is a strong inducer of type I IFNs, human cells infected with protoparvovirus H-1PV produce very little of these antiviral cytokines (65, 92). The Seneca Valley virus actively inhibits the production of type I IFN by cleaving adaptor proteins necessary for this process (93). Nevertheless, these various OVs all show promising oncosuppressive activity in preclinical and clinical studies, indicating that type I IFNs represent one of several factors that OVs can mobilize to heat up TMEs and activate immune responses against cancer cells.

In addition to the type I IFN induction resulting from some OV/tumor cell interactions, the way by which OVs kill cancer cells can stimulate an antitumor immune response before or during cancer cell lysis. Indeed, OVs (alone or in combination with cytotoxic agents) provoke various intracellular disturbances at the expense of cell organelles in particular mitochondria, lysosomes, endoplasmic reticulum and Golgi apparatus, which eventually results in the lysis of the cancer cell and the release of progeny viruses (94). Several OVs have been reported to induce oxidative stress with the production of ROS and reactive nitrogen species (RNS) and ER stress accompanied by Ca2+ release from ER with consequent Ca2+ dyshomeostasis and unfolded protein response (94). ROS/RNS may themselves induce ER stress with consequent Ca2+ release, while Ca2+ potentiates oxidative stress with enhanced production and release of ROS/RNS, thereby generating a positive amplification loop that results in the induction of apoptosis or other modes of cell death (95). Remarkably, OV-mediated cancer cell death is often immunogenic and associated with the expression, release, and/or exposure of DAMPs including ATP, high mobility group box 1 (HMGB1), and calreticulin (CRT). In particular, extracellular ATP acts as a “find me” signal promoting the recruitment of DCs (96), while HMGB1 functions as a danger signal ligand for Toll-like receptor 4 and can directly activate DCs (97). CRT exposure on the cell surface acts as an “eat me” signal neutralizing CD47 on tumor cells and promoting phagocytosis (98). DAMPs attract APCs, in particular DCs, into the TME and induce them to secrete inflammatory cytokines, present TAAs, and prime cytotoxic T-cells. The temporally concomitant release of type I IFNs and DAMPs from OV-infected tumor cells leads to the consideration of type I IFNs as DAMPs, because they trigger similar immunogenic effects and also because the expression of some DAMPs can most likely be activated by IFNs. While ATP, CRT, and HMGB1 represent the classical hallmarks of immunogenic cell death, other molecules behave as DAMPs, for instance annexin A1 (ANXA1) and cancer cell-derived nucleic acid (99). It would be interesting to analyze these molecules in the context of OV-induced cell death. Furthermore, it is most likely that other DAMPs involved in the completion of immunogenic cell death remain to be identified. Information about OV-mediated (immunogenic) tumor cell death is often incomplete and fragmentary (99) warranting further studies of this essential parameter of virotherapy. These studies will not only improve our understanding of the mode of action of OVs, but also provide clues to improve the efficacy of OV-based treatments.

It should also be stated that in addition to the above-mentioned effects on tumor and immune cells, some OVs are able to infect and replicate in endothelial cells. By causing disruption of tumor vessels, these OVs can thus contribute to the necrosis of tumor cells irrespective of their infection, through oxygen and nutrients deprivation (100–104). Furthermore, these OVs may also promote in this way the infiltration of immune cells into the TME.

**Figure 1:**
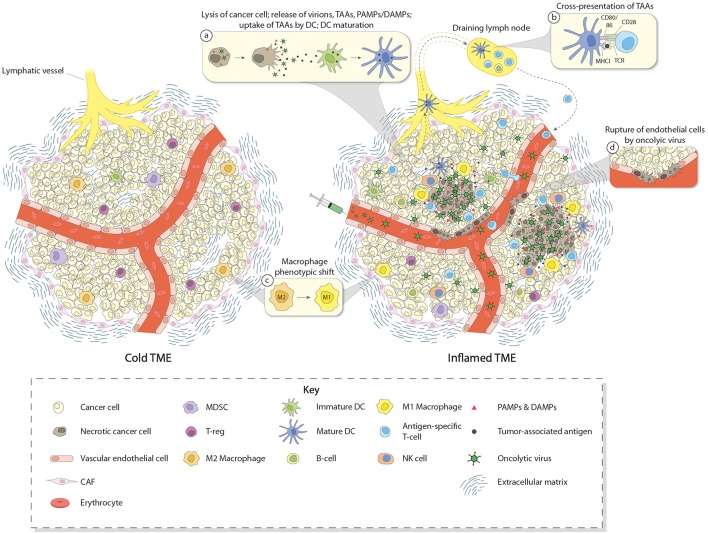
Induction of immune conversion of tumor microenvironment by OVs. The left panel depicts a cold tumor microenvironment (TME). In addition to tumor cells, some other components of the TME are shown, i.e., blood vessel with endothelial cells, CAFs, ECM, and few infiltrating immune cells. These immune cells (mainly Treg, MDSC, and TAM having a M2 immunosuppressive status), together with other cells of TME (e.g., CAF and tumor cells themselves), produce and secrete chemo/cytokines, growth factors, and other molecules which contribute to create an immunosuppressive TME. This “cold” TME supports tumor development and metastasis, and confers resistance to (immuno) therapies. The right panel depicts an inflamed TME after intravenous OV treatment. OVs reach the tumor through the blood stream and act in a multimodal fashion to eliminate cancer cells. OVs specifically replicate in and kill cancer cells by inducing immunogenic cell death. Virus-induced cancer cell lysis is associated with the release of progeny virus particles, TAAs, DAMPs, PAMPs, and pro-inflammatory/immunostimulatory cytokines which contribute to recruiting immune cells in the TME and inducing maturation of DCs, thereby triggering innate as well as adaptive immune responses (inset a). DCs migrate to the draining lymph nodes where they cross-present TAAs to T cells (inset b). After expansion, T cells infiltrate the TME and participate in the destruction of cancer cells together with other effector cells such as NK cells and M1-converted macrophages (inset c). Some OVs may also infect endothelial cells and induce disruption of tumor vasculature, potentially facilitating immune cell migration into the TME (inset d).

In summary, the great interest raised by OVs in the field of cancer therapy relies on their abilities (i) to specifically replicate, multiply, and spread in a lytic manner in tumor cells (oncolysis), (ii) to trigger the release of PAMPs and TAAs/TANs from dying tumor cells, leading to the activation of innate as well as adaptive immune responses, (iii) to directly induce the expression of pro-inflammatory and immuno-stimulatory cytokines, in particular type I IFNs, in some tumor and immune cells, and (iv) to kill tumor cells via immunogenic mechanisms (immunogenic cell death) involving the production of DAMPs that are able to further stimulate immune cells, and (v) for some OVs, to break-down tumor vasculature, causing tumor cell starvation and facilitating immune cell infiltration. Based on these considerations, OVs are attractive candidates to activate innate and adaptive immune responses in TMEs and turn immune-excluded or immune-deserted tumors into inflamed ones (Figure 1).

## Strategies to Improve OVs and Optimize Their Immune Stimulatory Activities

Despite promising results obtained at the preclinical level, only a small proportion of cancer patients seems to benefit from OV-mediated therapy in clinical studies. A number of reasons account for these disappointing results. OVs, especially when delivered systemically, need to overcome several physiological and physical barriers to reach the tumor target and be effective [reviewed in Marchini et al. (105)]. For instance, sequestration, and neutralization by the mononuclear phagocyte system can dramatically restrict the systemic delivery of OVs. The presence of specific neutralizing antibodies (NAb), can also severely hamper OV systemic delivery and effectiveness, especially in the case of OVs based on human pathogens to which patients may have been previously exposed. For instance, seroprevalence is high against Ad type 5 (60 and 70% in Europe and USA) (106) and HSV (50–80% worldwide) (107), two viruses commonly used as OVs. NAb recognize and coat the virus particles, signaling them for destruction by competent cells. Virus clearance can occur very rapidly, eliminating the virus before its anticancer potential is expressed. Optimal use of these OVs therefore requires consideration of the counteracting effect of pre-existing anti-viral immunity. However, recent results obtained with reovirus (against which about 80% of the human population has developed immunity) indicate that the presence of anti-viral NAb is not always a negative event and paradoxically, can even enhance the delivery of systemically administrated reovirus into the tumor bed. Indeed, NAb-reovirus complexes were found to be taken up and delivered to the tumor by the monocytes present in the blood (108, 109). In most cases, NAb still limit OV activity, as exemplified by a phase I trial demonstrating the greater efficiency of MV in myeloma patients devoid of pre-existing NAbs (110). Several strategies have been developed to overcome anti-viral host immune responses and improve delivery, e.g., virus capsid engineering (111), chemical modification of virus capsid [e.g., PEGylation (112)] and use of cell carriers (e.g., DCs) (113). For instance, Ad vector PEGylation was shown to reduce liver uptake, prevent NAb binding, and thereby improve Ad half-life in blood and infection of tumors (112).

Potentiation of OVs can be achieved by inserting (a) therapeutic transgene(s) into the viral genome. Notable examples are OVs armed with payloads that have immune stimulatory activity, such as pro-inflammatory cytokine (e.g., GM-CSF, IFN-γ, IL-2, IL-12, or IL-15) or chemokine (e.g., CCL2, CCL5, CCL19, CXCL11) transgenes. OV arming with cytokine and chemokine genes is aimed at providing additional stimuli for turning immune-excluded and deserted tumors into hot inflamed ones by induction of immune cell migration and activation. The impact of different arming strategies on tumor heating up has been recently reviewed by de Graaf et al. (114). The success of this approach is exemplified by the HSV-based T-Vec recombinant expressing GM-CSF, a cytokine that stimulates DC migration and maturation, thereby conferring the virus with enhanced capacity for inducing antigen presentation and T-cell priming (68). Intratumoral administration of T-Vec was found to induce the regression of not only injected tumors but also of non-injected distant tumors, including visceral metastases, indicating virus ability to trigger a systemic antitumor immune response (68, 115, 116). Furthermore, treated mice were protected from re-challenging with the same tumor cells, which indicate a durable antitumor memory response (117). However, it should be stated that cytokine arming, in the context of OV therapy, needs to be carefully evaluated on a case-by-case basis, considering that OVs are replication-competent and transgene expression may thus get amplified. Cytokine overexpression can be deleterious, as illustrated by the severe side effects and hepatic toxicity associated with high dose regimes of recombinant IL-2.

Another promising approach makes use of OVs expressing bi-specific T-cell engagers (BiTEs). BiTEs represent a new class of immunotherapeutic molecules which consist of two single-chain variable fragments (scFv) connected by a flexible linker. One scFv recognizes a T-cell-specific molecule, e.g., CD3, while the second scFv is directed against a TAA expressed on the surface of tumor cells. In this way, BiTEs lead T-cells to target tumor cells, ultimately stimulating T-cell activation, tumor cell killing, and cytokine production. In addition to exert their intrinsic anticancer activity, OVs expressing BITEs are thus able to mobilize T-cells at tumor sites, resulting in an increased oncosuppressive potential (118, 119).

Important improvements of anticancer efficacy have been achieved by inserting genes encoding for scFv targeting immune checkpoint molecules (e.g., PD-1 or CTLA-4) into the viral genome. This approach has been applied successfully with myxoma virus, Ad, MV, and VV (120–123). By achieving intra-tumoral delivery and expression of checkpoint blockade, these recombinant OVs alleviate the risk of systemic unspecific side effects often encountered when the antibody blockers are administered by intravenous infusion. As the PD-1/PD-L1 checkpoint control may be triggered by tumor cells, i.e., at the site of action of OVs, the synergism between the latter and PD-1/PD-L1 checkpoint blockade is expected to be most efficient for this particular immune checkpoint. Indeed, the oncosuppressive activity of MV was found to be reinforced to a greater extent by anti-PD-1/PD-L1 than anti-CTLA-4 transgenes (122). However, intratumorally produced CTLA-4-specific antibodies may still enhance the adaptive antitumor response triggered by OV-activated APCs by getting transported to draining lymph nodes or intratumoral tertiary lymphoid structures where priming takes place. Indeed, growing evidence supports the assumption that local delivery of CTLA-4 blockade can trigger T-cell priming in the periphery (124) and release local effector cells by depleting intratumoral Tregs (125).

Combining multiple OVs also opens up interesting prospects, as demonstrated by a recent study in which Ad treatment was followed by VV administration in a Syrian hamster model. The first line OV treatment was found to protect the second virus from the attack of the immune system, enlarging its therapeutic window, and enhancing efficacy (126). An especially intriguing approach consists in a prime-boost protocol involving the sequential application of two distinct oncolytic viruses (vectors): a first one for priming the immune-system to recognize TAAs and a second one for boosting this response through virus-mediated TAA expression after systemic OV administration [reviewed in (127, 128)]. Also in this case, the use of a different virus vector in the priming phase may reduce the insurgence of NAb against the second virus used during the boosting phase. This strategy has also the potential to sensitize tumors to checkpoint blockade (129).

OV therapy is also compatible with other anticancer modalities, and investigation of OV-based combination treatments is actively being pursued with all OVs under clinical development.

- For the sake of expediting clinical translation, OV administration has been combined with conventional chemotherapy and radiotherapy, resulting in a number of cases in synergistic anticancer effects at the preclinical level (67, 130, 131). Some of these combinations are currently being tested in clinical studies.
- In addition to combining with standard treatments, OVs are being tested together with immunomodulators, including drugs that induce immunogenic cell death [reviewed in (94)] or dampen the antiviral innate response (3).
- A particularly promising area of active research involves the combination of OVs with adoptive immune cell therapy. In particular, OV can improve the efficacy of CAR-modified T cell transfer therapy [as recently reviewed in (3, 132)].
- Owing to their ability to induce immune conversion of TMEs, a number of OVs have been clinically tested in combination with checkpoint blockade against a broad range of malignancies [for a recent review see (133)]. The joint application of these therapies is anticipated to improve the clinical outcome in cancer patients by eliciting more robust anticancer immune responses. This concept is supported by multiple preclinical evidence showing that OV treatment sensitizes tumors to checkpoint blockade, resulting in synergistic anticancer activity in animal models (122, 134–139). Recent studies by Samson et al. (138) provided further appealing clinical evidence of OV ability to convert a cold tumor previously resistant to immune checkpoint blockade therapy, into a hot tumor sensitive to immune therapy. This conversion may be due in part to the OV-induced local expression of type I IFNs and type II IFN-γ resulting in the up-regulation of inhibitory ligands (PD-L1 and PD-L2) on tumor cells (140) and thereby making “cold” tumors susceptible to immune checkpoint blockade. Upon intravenous treatment with reovirus, patients with high-grade glioma, or brain metastases, showed increased intratumoral leukocyte infiltration and type I and II IFN-dependent induction of PD-L1 expression (138).

OV-mediated potentiation of checkpoint blockade immunotherapy was also demonstrated in clinical studies using T-Vec in combination with ipilimumab (anti CTLA-4) or pembrolizumab (anti PD-1) (68). In particular, in a randomized open-label phase II trial involving 198 patients with advanced melanoma, ipilimubab/T-Vec co-treatment produced higher objective response rates (ORR) (39 vs. 18%), with 89% of all co-treated patients experiencing durable responses at a median follow up time of 16 months. Furthermore, 52% of patients presented reduced visceral lesions, providing evidence that T-Vec enhanced systemic antitumor immune responses (141). Promising results were also obtained by combining T-Vec with pembrolizumab in a trial's phase Ib arm involving 21 patients with advanced melanoma. In this trial, co-treated patients showed a higher ORR (62%), compared with patients treated with pembrolizumab (34%) or T-Vec (26%) alone. Immune conversion of the TME was observed in co-treated patients, including CD8+ T cell infiltration and both elevated PD-L1 protein expression levels and IFN-γ production in tumor cells (142). It should also be stated that co-treatment was not associated with additional toxicity. Extension of this trial is ongoing and involves a total of 660 patients receiving either combination treatment or pembrolizumab alone (68).

The pros and cons of oncolytic virotherapy and the current attempts at improving this strategy will be exemplified in the following section with one of the OVs in clinical development, the rodent protoparvovirus H-1PV.

## The Rat Protoparvovirus H-1PV

### The Virus

The oncolytic protoparvovirus (PV) H-1PV is a non-enveloped single-stranded DNA virus (143, 144). With an icosahedral capsid of 25 nm, H-1PV is the smallest OV presently under clinical development. H-1PV belongs to the *Parvoviridae* family, genus *Protoparvovirus*, species *Rodent protoparvovirus 1*. The *Parvoviridae* family also includes adeno-associated viruses (AAV) that are commonly used in gene therapy for the delivery of therapeutic transgenes (143, 144). However, in contrast to AAVs which need a helper virus for their replication, H-1PV as other protoparvoviruses can replicate autonomously. The *Rodent protoparvovirus 1* also includes the Kiham rat virus, LuIII virus, mouse parvovirus, minute virus of mice (MVM), tumor virus X, and rat minute virus. Some of these viruses are under evaluation at the preclinical level as oncolytic agents.

The H-1PV genome comprises ~5,100 nucleotides. Small deletions and point mutations can naturally occur in the parvoviral genome, reflecting genetic adaptation to the molecular characteristics of the host cell. The genome consists of two transcription units, termed NS and VP, whose expression is controlled by the early (P4) and late (P38) promoters, respectively. The NS gene unit encodes the non-structural proteins NS1, NS2, and NS3 while the VP unit encodes the VP1, VP2/VP3 capsid proteins and the non-structural SAT protein.

The natural host of H-1PV is the rat; the virus is not pathogenic to humans. H-1PV is unable to replicate in normal tissues, but it can productively infect and kill a broad range of human cancer cell lines from different origins including glioma, breast cancer, hepatoma, pancreatic carcinoma, melanoma, colorectal carcinoma, nasopharyngeal carcinoma, and lymphoma (143). H-1PV oncosuppression has been demonstrated in a number of preclinical animal models (143).

The reasons for H-1PV intrinsic oncotropism and tumor selectivity have been elucidated only in part and are discussed in detail elsewhere (143–146). In brief, the virus has the ability to exploit some of the molecular features that distinguish the cancer cell, such as (i) fast proliferation associated with the overexpression and/or activation of specific cellular factors needed for virus DNA replication and gene transcription belonging to the E2F, ATF/CREB, ETS, NFY families and cyclin A, and (ii) altered signaling pathways accompanied by upregulation of factors controlling viral functions (e.g., the PDK1/PKB/PKC pathway involved in the phosphorylation of the oncotoxic viral protein NS1); (iii) impairment of the innate antiviral immune response in many tumor cells, although the sensitivity of rodent PVs to type I IFN is presently a matter of controversy (92, 144, 147–150).

### H-1PV, an Oncolytic Virus Case in Point

Although underlying mechanisms may be at least partly different, H-1PV, and various other OVs share a number of properties that illustrate well the pros and cons of cancer virotherapy. Pros comprise safety, oncotropism, oncosuppressive ability resulting from both oncolytic and immune adjuvant properties, and the possibility of systemic administration. Cons include limited tumor capacity for virus production in cancer patients, and inter/intratumoral heterogeneity of cancer cell permissiveness for virus infection. H-1PV can thus be used to exemplify the prospects and drawbacks of oncolytic virotherapy. PVs still have a number of unique properties distinguishing them from several other OVs [for a review, see Geletneky et al. (151)]. On the one hand, their lack of natural infectiousness and pathogenicity for humans, and the synthetic oncotoxicity of the viral protein NS1 are worth mentioning. On the other hand, PVs are taken up by most normal cells, which leads to typically harmless abortive infections, but results in the sequestration of a major fraction of administered/produced virions in normal tissues. This trapping limits the capacity of H-1PV to work at a distance from the inoculation site, making viral remote activity especially dependent on a bystander immune adjuvant effect, which is the focus of the present review.

### H-1PV Oncolytic Activity

Besides being the key regulator of H-1PV replication, the NS1 protein is the major effector of virus oncotoxicity. The molecular mechanisms underlying H-1PV-mediated cell death are not fully understood. It was demonstrated that through NS1, H-1PV has the ability to induce oxidative stress associated with elevated levels of intracellular ROS, RNS, and DNA damage resulting in the activation of the intrinsic pathway of apoptosis (152).

In addition to apoptosis, the virus can activate a range of other cell death programs, including necrosis and cathepsin-mediated cell death in glioma cells (143). The latter mechanism involves relocation of active cathepsins from lysosomes into the cytoplasm accompanied by the downregulation of cystatin B and C, two cathepsin inhibitors (153). In support of the capacity of H-1PV to induce lysosomal-mediated necrosis in glioma cells, we recently obtained evidence of the occurrence of lysosomal membrane permeabilization and ER stress after infection of these cells (Marchini et al. unpublished results). The SAT protein may have a role in H-1PV-mediated ER stress as observed for porcine parvovirus (PPV). A PPV mutant with a deletion in the SAT region had less lytic activity than the wild-type virus and consequently less spreading (154). It is therefore possible that SAT together with NS1 participates in H-1PV-mediated cell death, in agreement with recent results from our laboratory (Bretscher et al. unpublished results).

Depending on the characteristics of the target tumor and on the amount of virus penetrating the tumor cell (i.e., input virus dose), it is possible that multiple cell death pathways are activated in parallel. It is important to point out that some forms of cell death may be more immunogenic than others and may therefore influence the outcome of H-1PV-based therapies by engaging the immune system at different levels. This needs to be carefully considered in the design of therapeutic protocols (e.g., effective viral dose and treatment fractionation), especially in the context of combination regimens.

The extent of H-1PV-induced oxidative stress may account in part for the capacity of the virus to induce different types of cell death. Indeed, it has been demonstrated that intracellular ROS/RNS levels are pivotal for the determination of cell fate by fine-tuning cell stress responses. While physiological levels of ROS promote cell proliferation, excess production/accumulation of these toxic compounds has been associated with DNA damage and major disturbances such as activation of the inflammasome, induction of TNF-mediated inflammatory pathways, lipid peroxidation, lysosomal dysfunction, ER stress, and calcium and iron dyshomeostasis. Depending on the genetic background of the cell, different ROS/RNS levels can activate distinct forms of cell death, such as apoptosis, pyroptosis, necroptosis, ferroptosis, authophagy, and necrosis (95, 99). Some cancer cells may have more efficient antioxidant mechanisms to counteract H-1PV-induced oxidative stress and therefore be less susceptible to virus oncotoxicity. However, through its ability to activate different cell death pathways, H-1PV may compensate for cancer cell resistance to apoptotic stimuli or DNA damage-inducing agents by engaging the immune system to act against the tumor. Indeed, as briefly summarized in the next section, there is accumulating evidence supporting a role for H-1PV as an activator of immune-mediated anticancer responses.

### H-1PV-Mediated Immune Modulation

Preclinical studies of H-1PV demonstrated the involvement of multiple immune cell populations in the anti-neoplastic activity of this virus. Distinct immune cells proved to be activated by H-1PV as a result of both their direct infection with the virus and their exposure to virus-induced tumor cell lysates.

H-1PV can infect a wide panel of human immune cells, namely DCs, macrophages, NK cells, and T-lymphocytes. Infection is abortive and does not result in the production of progeny viral particles. More importantly, no or little direct toxicity of H-1PV for human immunocytes has been observed, while the induced release of cytokines may cause cytopathic effects under *in vitro* conditions (155, 156). H-1PV has been shown to be harmless for rat immune cells as well. Rats treated with repeated high doses of H-1PV showed normal activity of B-cells and developed NAbs against H-1PV. Serum concentrations of IL-6 and TNF-α were normal in these animals, and isolated PBMCs showed proliferative response similar to control (157).

H-1PV infection of human PMBCs results in their maturation and activation, which are associated with the release of IFN-γ and TNF-α. Furthermore, a type I IFN production mediated at least in part by TLR-9 was observed and assigned to infected plasmacytoid DCs (156). Interestingly, H-1PV infection proved able to stimulate CD4+ T-cells, as revealed by the enhanced expression of activation markers (CD69 and CD30) and release of both Th1 and Th2 cytokines (IL-2, IFN-γ, and IL-4) (158).

In addition to its direct impact on human immune cells, H-1PV indirectly causes major immune stimulatory effects which are apparently induced by infected cancer cells. Indeed, while failing to induce type I IFN in these cells, H-1PV can indirectly upregulate both innate and adaptive immune responses through its effects on tumor cells.

On the innate side, H-1PV infection of human pancreas and colon carcinoma cells was shown to enhance their ability to stimulate NK cells, as a result of the downregulation of MHC-I molecules and upregulation of NK-activating ligands on the surface of infected tumor cells. This stimulation is reflected in an increase of both the release of cyto/chemokines (IFN-γ, TNF-α, and MIP-1), and the killing of tumor cells by NK cells (159).

On the adaptive side, effector Th cells (with a Th1 bias) were found to be stimulated in the presence of H-1PV-infected tumor cells, at least in part through the enhanced capacity of the latter for activating APCs. Infection of pancreatic ductal adenocarcinoma (PDAC) cells with H-1PV leads to the release of HMGB1 but apparently not CRT or ATP (160). As mentioned above, HMGB1 interacts with TLR4 and can directly activate DCs. Furthermore, infection of human melanoma cells with H-1PV induces them to release HSP72. Extracellular HSP72 has potent adjuvant properties and can induce migration and activation of DCs as well as activation of NK cells (155, 161). H-1PV-infected melanoma cell lysates are indeed able to induce maturation of DCs, as revealed by the upregulation of co-stimulatory molecules (CD86) and the production of pro-inflammatory cytokines (TNF-α and IL-6) (162). The maturation of DCs resulting from their incubation with H-1PV-induced melanoma cell lysates correlates with the up-regulation of TLR3 and TLR9 expression and the activation of the NFκB signaling pathway (163). DCs pulsed with lysates of H-1PV-infected tumor cells not only mature and produce pro-inflammatory cytokines, but also show the ability to cross-present TAAs to specific CTLs, linking the stimulation of innate immunity to the activation of an adaptive immune response (164).

H-1PV requires functional adaptive immunity to fully express its therapeutic potential. CD8+ cells are essential to suppress metastases of Morris hepatoma cells in rats treated with a therapeutic vaccine based on H-1PV-infected autologous tumor cells (165). Similarly, antibody depletion of CD8+ cells in an immunocompetent rat model of glioma, strongly diminished H-1PV oncosuppressive activity (166). Immune reconstitution of NOD SCID mice bearing human PDAC transplants with autologous DC and T-cells primed *ex vivo* with H-1PV-induced tumor cell lysates resulted in a strong suppression of tumor development (167). These data directly demonstrate the adjuvant effect of H-1PV on the efficacy of a cancer vaccine. It is noteworthy that the vaccination potential of H-1PV can be further improved by combination therapy with IFN-γ (168). The involvement of CTLs in H-1PV anti-cancer activity was further demonstrated in a rat syngeneic bilateral PDAC model. Rats engrafted with tumors in both flanks and injected with virus in only one site, experienced significant reduction in tumor size at both the injected and the distal uninjected sites, arguing for an involvement of the immune system in the regression of untreated lesions. H-1PV particles were not detected in uninjected tumors which instead showed increased expression of IFN-γ, granzyme B and perforin (169). As additional proof of the role of CTLs in the therapeutic activity of H-1PV, adoptive transfer of splenocytes from H-1PV-treated donors into naïve recipients was shown to significantly prolong survival of animals harboring PDAC (167).

### H-1PV Clinical Development

H-1PV is one of the OVs that have successfully transitioned from preclinical studies into clinical development. Two clinical trials, in brain and gastrointestinal (pancreatic) tumor patients, have been conducted recently.

#### Glioblastoma

Glioblastoma is recognized as one of the tumors with the “coldest” TME. Infiltration of immune cells into the glioblastoma bed is generally very limited (170). Furthermore, mutational signature studies in glioblastoma have revealed the presence of only 30–50 non-synonymous mutations (171). As mentioned above, the success of antigen-specific immunotherapies, such as checkpoint blockade, largely depends on tumor mutational load and the presence and phenotype of tumor-infiltrating immune cells. The benefit of this approach for patients with glioblastoma is therefore presently insufficient and badly predictable. In contrast to this intrinsically low responsiveness to checkpoint blockade seen in the majority of glioblastoma patients, H-1PV treatment of the latter is unlikely to be compromised by the “cold” TME. Moreover, H-1PV-induced tumor cell killing, DAMP/PAMP release and increased neoantigen exposure [recently reviewed in Angelova and Rommelaere (150)] may contribute to TME “warming up” and not only trigger antitumor immune responses *per se*, but also alleviate glioblastoma resistance to checkpoint inhibition. Based on the above, H-1PV deserves consideration also as partner drug in combinatorial immune checkpoint blockade treatments directed against glioblastoma and other tumors with low mutational load.

Similar concerns apply to the applicability of CAR T-cell therapy in glioblastoma. Currently, three CAR T-cell trials have been published which reported promising signs of efficacy in selected glioblastoma patients (172). However, also here the immunosuppressive glioblastoma TME presents obstacles and poses barriers to CAR T-cell proliferation and responses. Whether administered as preceding treatment or simultaneously, in combination with CAR T-cells, H-1PV-mediated TME immune stimulation holds the promise for synergizing with CAR T-cell efficiency. H-1PV-based combinatorial approaches which have yielded encouraging evidence of preclinical and clinical efficacy were recently reviewed in Bretscher and Marchini (144) and are briefly listed below (see Future perspectives in PV therapeutic development).

Yet another advantage of H-1PV as anticancer immunomodulator lies in the gentle way in which the virus reshapes the TME and boosts the immune system. Contrary to immune checkpoint blockade- and CAR T-cell therapy-associated organ toxicities and immune-related adverse events, H-1PV administration to glioblastoma patients is not accompanied by any signs of immune system overstimulation and does not exert any negative impact on laboratory safety parameters. Furthermore, no dose-limiting toxicity could be reached in the first parvovirus glioblastoma clinical trial, as described in more detail below.

The preclinical proof of concept for H-1PV-based virotherapy of brain tumors was provided by *in vivo* experimental evidence demonstrating efficient H-1PV-induced suppression of both rat and human gliomas in syngeneic or immunodeficient animal models, respectively (173). Progressive reduction of tumor size, complete remission in 50% of the responding animals and significant survival prolongation were observed, while no H-1PV treatment-associated side effects could be detected. These data paved the way for the launch of the first-in-man PV clinical trial (ParvOryx01), a phase I/IIa study in patients with recurrent glioblastoma (174). Notably, ParvOryx01 was also the first OV trial in Germany (175). Within the frame of the trial, 18 patients with a history of one previous glioblastoma resection were treated with escalating H-1PV (GMP-grade, ParvOryx) doses. Half of the corresponding dose was applied either intratumorally or intravenously before tumor resection. After tumor resection, at day 10 after treatment, the second half of the planned virus dose was injected into the wall of the resection cavity. The primary trial endpoints were safety, tolerability, pharmacokinetics, and maximum tolerated dose (MTD) estimation. In addition, tumor tissue samples were acquired during resection, allowing for the analysis of markers of intratumoral virus expression and TME immunological landscape. ParvOryx01 convincingly proved H-1PV safety and tolerability (176). MTD could not be reached. Risk assessment ruled out virus transmission from study patients to third persons, since no infectious H-1PV particles were found in fecal and urine samples. Analysis of post-treatment tumor tissues detected virus expression in a subset of glioblastoma cells and remarkably, also in those patients who received systemic ParvOryx treatment. This was in line with preclinical reports showing H-1PV ability to cross the blood-brain/tumor barrier after intravenous administration. Furthermore, TME immune conversion was observed (176). ParvOryx treatment promoted tumor infiltration with immune cells. Most of the infiltrate consisted of Th cells and perforin- and granzyme B-expressing CTLs. Of note, only scarce Treg cells were seen scattered within the tumor. Activation of glioblastoma-associated microglia/macrophages and detection of pro-inflammatory cytokine production in treated tumors hinted at the induction of an inflamed microenvironment and increased immunological visibility of the tumor. Interestingly, in some of the study patients, formation of not only virus-specific but also glioma-specific T-cell responses was demonstrated, raising the hope that H-1PV treatment may contribute to the circumvention of tumor immune evasion mechanisms in glioblastoma and other poorly immunogenic human tumors.

#### Pancreatic Ductal Adenocarcinoma (PDAC)

The second H-1PV clinical trial (ParvOryx02) was launched in 2015 in patients with metastatic inoperable pancreatic cancer (177). ParvOryx02 was recently successfully completed and clinical and research findings are currently awaiting publication.

### Future Perspectives in PV Therapeutic Development

The first clinical evidence of H-1PV capacity to induce an inflamed TME in glioblastoma patients together with favorable survival data (176, 178), prompted further efforts to develop strategies to increase the efficiency of PV-based cancer viro(immuno)therapy. Several approaches hold particular promise and are currently under investigation.

H-1PV-based combinatorial treatments have been evaluated in both preclinical and clinical settings (144). H-1PV combinations with chemotherapeutics (160, 179), histone deacetylase (HDAC) inhibitors such as valproic acid (VPA) (180) and immune checkpoint blockade (181) have been demonstrated to synergistically potentiate the double-faceted anticancer activity of the virus by both inducing enhanced virus replication, oxidative stress and tumor cell lysis (180), and exerting immune stimulatory effects (160, 181) in tumor cell and animal models. Notably, some H-1PV-based combinatorial approaches have also been tested in the clinic. The ParvOryx02 trial combined systemic and intramethastatic H-1PV administration with gemcitabine, the gold standard first-line therapy for PDAC patients. Within the frame of a compassionate use program, favorable response was achieved in glioblastoma patients treated with H-1PV and bevacizumab, an anti-angiogenic agent with still underappreciated immunomodulating properties (182). Some of the patients were also co-treated with the PD-1 inhibitor nivolumab and based on the positive results obtained at the preclinical level, with VPA. This multimodal treatment resulted in partial or complete objective responses in 7 of 9 cases (183, 184). These encouraging results strongly support further (pre)clinical development of PV-based viro(immuno)/chemotherapies for glioblastoma and other cancers treatment.

### H-1PV Genetic Engineering

Another intriguing approach to PV efficacy potentiation is arming the PV genome with immunostimulatory CpG motifs (185) or therapeutic transgenes encoding for angiostatic/immunostimulatory molecules (186). However, in the latter example, due to the limited packaging capacity of H-1PV, the therapeutic transgene replaces part of the viral genomic region encoding for the capsid proteins, rendering the virus replication deficient. Production of these recombinant PVs requires the use of helper plasmids (187, 188).

The limited packaging capacity of H-1PV can be overcome through an original strategy proposed by El-Andaloussi et al. (189). An engineered H-1PV genome is inserted into the genome of a replication-defective Ad5 vector. The resulting chimera not only allows H-1PV genome delivery to cancer cells with subsequent production and release of infectious replication-competent viral particles but, importantly, also offers new prospects for reinforcing the anticancer activity of H-1PV by inserting a therapeutic gene into the adenovirus component of the Ad-PV hybrid genome. This chimera provides a unique platform to carry out, by means of a single agent, cancer gene therapy (through the replication-deficient transgene-armed adenovirus carrier) and oncolytic virotherapy (through the released replication-competent H-1PV particles).

As for every OV under investigation and more generally for any other anticancer agents, further development of H-1PV-based therapies would certainly benefit from the establishment of novel models (e.g., use of patient-derived spheroids/organoids, syngeneic, or humanized animal models) that more closely recapitulate human disease and better predict the outcome of the novel therapies once transferred to the clinic.

## Author Contributions

AM, LD, VP, AA, and JR have contributed in the writing of the manuscript, read and approved the final manuscript. AM designed the figure.

### Conflict of Interest Statement

AM, LD, AA, and JR are inventors in several H-1PV-related patents/patent applications. The remaining author declares that the research was conducted in the absence of any commercial or financial relationships that could be construed as a potential conflict of interest.

## References

[1] Ledford H. Cancer-fighting viruses win approval. Nature. (2015) 526:622–3. 10.1038/526622a 26511559

[2] Kaufman HL Kohlhapp FJ Zloza A Oncolytic viruses: a new class of immunotherapy drugs Nat Rev Drug Discov. (2015) 14:642–62. 10.1038/nrd4663 26323545 PMC7097180

[3] Achard C Surendran A Wedge ME Ungerechts G Bell J Ilkow CS. Lighting a fire in the tumor microenvironment using oncolytic immunotherapy. EBioMed. (2018) 31:17–24. 10.1016/j.ebiom.2018.04.020 29724655 PMC6013846

[4] Martinez-Quintanilla J Seah I Chua M Shah K. Oncolytic viruses: overcoming translational challenges. J Clin Invest. (2019) 130:1407–18. 10.1172/JCI122287 30829653 PMC6436848

[5] Balkwill FR Capasso M Hagemann T. The tumor microenvironment at a glance. J Cell Sci. (2012) 125:5591–6. 10.1242/jcs.116392 23420197

[6] Najafi M Goradel NH Farhood B Salehi E Solhjoo S Toolee H. Tumor microenvironment: interactions and therapy. J Cell Physiol. (2019) 234:5700–21. 10.1002/jcp.27425 30378106

[7] Poltavets V Kochetkova M Pitson SM Samuel MS. The role of the extracellular matrix and its molecular and cellular regulators in cancer cell plasticity. Front Oncol. (2018) 8:431. 10.3389/fonc.2018.00431 30356678 PMC6189298

[8] Simi AK Pang FM Nelson CM. Extracellular matrix stiffness exists in a feedback loop that drives tumor progression. Adv Exp Med Biol. (2018) 1092:57–67. 10.1007/978-3-319-95294-9_4 30368748

[9] Rossing M Sørensen CS Ejlertsen B Nielsen FC. Whole genome sequencing of breast cancer. APMIS. (2019) 127:303–15. 10.1111/apm.12920 30689231 PMC6850492

[10] Kondou R Iizuka A Nonomura C Miyata H Ashizawa T Nagashima T. Classification of tumor microenvironment immune types based on immune response-associated gene expression. Int J Oncol. (2019) 54:219–28. 10.3892/ijo.2018.4617 30387832

[11] De Palma M Biziato D Petrova TV. Microenvironmental regulation of tumour angiogenesis. Nat Rev Cancer. (2017) 17:457–74. 10.1038/nrc.2017.51 28706266

[12] Becker JC Andersen MH Schrama D Thor Straten P. Immune-suppressive properties of the tumor microenvironment. Cancer Immunol Immunother. (2013) 62:1137–48. 10.1007/s00262-013-1434-6 23666510 PMC11029603

[13] Durgeau A Virk Y Corgnac S Mami-Chouaib F. Recent advances in targeting CD8 T-cell immunity for more effective cancer immunotherapy. Front Immunol. (2018) 9:14. 10.3389/fimmu.2018.00014 29403496 PMC5786548

[14] Molodtsov A Turk MJ. Tissue resident CD8 memory T cell responses in cancer and autoimmunity. Front Immunol. (2018) 9:2810. 10.3389/fimmu.2018.02810 30555481 PMC6281983

[15] Souza-Fonseca-Guimaraes F Cursons J Huntington ND. The emergence of natural killer cells as a major target in cancer immunotherapy. Trends Immunol. (2019) 40:142–58. 10.1016/j.it.2018.12.003 30639050

[16] Shen J Xiao Z Zhao Q Li M Wu X Zhang L. Anti-cancer therapy with TNFα and IFNγ: a comprehensive review. Cell Proliferation. (2018) 51:e12441. 10.1111/cpr.12441 29484738 PMC6528874

[17] O'Higgins C Ward FJ Abu Eid R. Deciphering the role of regulatory CD4 T cells in oral and oropharyngeal cancer: a systematic review. Front Oncol. (2018) 8:442. 10.3389/fonc.2018.00442 30460193 PMC6232931

[18] Conlon KC Miljkovic MD Waldmann TA. Cytokines in the treatment of cancer. J Interfer Cytok Res. (2019) 39:6–21. 10.1089/jir.2018.0019 29889594 PMC6350412

[19] Taube JM Klein A Brahmer JR Xu H Pan X Kim JH. Association of PD-1, PD-1 ligands, and other features of the tumor immune microenvironment with response to anti-PD-1 therapy. Clin Cancer Res. (2014) 20:5064–74. 10.1158/1078-0432.CCR-13-3271 24714771 PMC4185001

[20] Herbst RS Soria JC Kowanetz M Fine GD Hamid O Gordon MS. Predictive correlates of response to the anti-PD-L1 antibody MPDL3280A in cancer patients. Nature. (2014) 515:563–7. 10.1038/nature14011 25428504 PMC4836193

[21] Munn DH Bronte V. Immune suppressive mechanisms in the tumor microenvironment. Curr Opin Immunol. (2016) 39:1–6. 10.1016/j.coi.2015.10.009 26609943 PMC5627973

[22] Fearon DT Immune-suppressing cellular elements of the tumor microenvironment. Ann Rev Cancer Biol. (2017) 1:241–55. 10.1146/annurev-cancerbio-050216-034359

[23] Liu T Zhou L Li D Andl T Zhang Y. Cancer-associated fibroblasts build and secure the tumor microenvironment. Front Cell Dev Biol. (2019) 7:60. 10.3389/fcell.2019.00060 31106200 PMC6492564

[24] Lakins MA Ghorani E Munir H Martins CP Shields JD. Cancer-associated fibroblasts induce antigen-specific deletion of CD8 (+) T Cells to protect tumour cells. Nat Commun. (2018) 9:948. 10.1038/s41467-018-03347-0 29507342 PMC5838096

[25] Correa LH Correa R Farinasso CM de Sant'Ana Dourado LP Magalhaes KG. Adipocytes and macrophages interplay in the orchestration of tumor microenvironment: new implications in cancer progression. Front Immunol. (2017) 8:1129. 10.3389/fimmu.2017.01129 28970834 PMC5609576

[26] Saleh R Elkord E. Treg-mediated acquired resistance to immune checkpoint inhibitors. Cancer Lett. (2019) 457:168–79. 10.1016/j.canlet.2019.05.003 31078738

[27] Yano H Andrews LP Workman CJ Vignali DAA. Intratumoral regulatory T cells: markers, subsets and their impact on anti-tumor immunity. Immunology. (2019) 157:232–47. 10.1111/imm.13067 31087644 PMC6587321

[28] Rosser EC Mauri C. Regulatory B cells: origin, phenotype, and function. Immunity. (2015) 42:607–12. 10.1016/j.immuni.2015.04.005 25902480

[29] Sarvaria A Madrigal JA Saudemont A. B cell regulation in cancer and anti-tumor immunity. Cell Mol Immunol. (2017) 14:662–74. 10.1038/cmi.2017.35 28626234 PMC5549607

[30] Consonni FM Porta C Marino A Pandolfo C Mola S Bleve A. Myeloid-derived suppressor cells: ductile targets in disease. Front Immunol. (2019) 10:949. 10.3389/fimmu.2019.00949 31130949 PMC6509569

[31] Gabrilovich DI Ostrand-Rosenberg S Bronte V. Coordinated regulation of myeloid cells by tumours. Nat Rev Immunol. (2012) 12:253–68. 10.1038/nri3175 22437938 PMC3587148

[32] Cheng H Wang Z Fu L Xu T. Macrophage polarization in the development and progression of ovarian cancers: an overview. Front Oncol. (2019) 9:421. 10.3389/fonc.2019.00421 31192126 PMC6540821

[33] Visan I. New ligand for LAG-3. Nat Immunol. (2019) 20:111. 10.1038/s41590-018-0307-8 30664758

[34] Anderson AC Joller N Kuchroo VK. Lag-3, Tim-3, and TIGIT: Co-inhibitory receptors with specialized functions in immune regulation. Immunity. (2016) 44:989–1004. 10.1016/j.immuni.2016.05.001 27192565 PMC4942846

[35] Pardoll DM. The blockade of immune checkpoints in cancer immunotherapy. Nat Rev Cancer. (2012) 12:252–64. 10.1038/nrc3239 22437870 PMC4856023

[36] Dyck L Mills KHG. Immune checkpoints and their inhibition in cancer and infectious diseases. Eur J Immunol. (2017) 47:765–79. 10.1002/eji.201646875 28393361

[37] Wennerberg E Vanpouille-Box C Bornstein S Yamazaki T Demaria S Galluzzi L. Immune recognition of irradiated cancer cells. Immunol Rev. (2017) 280:220–30. 10.1111/imr.12568 29027232 PMC5659195

[38] Wei SC Duffy CR Allison JP. Fundamental mechanisms of immune checkpoint blockade therapy. Cancer Discov. (2018) 8:1069–86. 10.1158/2159-8290.CD-18-0367 30115704

[39] Dyck L Mills KHG. Immune checkpoints and their inhibition in cancer and infectious diseases. Eur J Immunol. (2017) 47:765–79. 28393361 10.1002/eji.201646875

[40] Couzin-Frankel J. Breakthrough of the year 2013. Cancer Immunother Sci. (2013) 342:1432–3. 10.1126/science.342.6165.1432 24357284

[41] Fennemann FL de Vries IJM Figdor CG Verdoes M. Attacking tumors from all sides: personalized multiplex vaccines to tackle intratumor heterogeneity. Front Immunol. (2019) 10:824. 10.3389/fimmu.2019.00824 31040852 PMC6476980

[42] Zhang H Chen J. Current status and future directions of cancer immunotherapy. J Cancer. (2018) 9:1773–81. 10.7150/jca.24577 29805703 PMC5968765

[43] Koury J Lucero M Cato C Chang L Geiger J Henry D. Immunotherapies: exploiting the immune system for cancer treatment. J Immunol Res. (2018) 2018:9585614. 10.1155/2018/9585614 29725606 PMC5872614

[44] D'Errico G Machado HL Sainz B. A current perspective on cancer immune therapy: step-by-step approach to constructing the magic bullet. Clin Transl Med. (2017) 6:3. 10.1186/s40169-016-0130-5 28050779 PMC5209322

[45] Sabado RL Balan S Bhardwaj N. Dendritic cell-based immunotherapy. Cell Res. (2017) 27:74–95. 10.1038/cr.2016.157 28025976 PMC5223236

[46] Ajina A Maher J. Prospects for combined use of oncolytic viruses and CAR T-cells. J Immunother Cancer. (2017) 5:90. 10.1186/s40425-017-0294-6 29157300 PMC5696728

[47] Yang J Hu L. Immunomodulators targeting the PD-1/PD-L1 protein-protein interaction: From antibodies to small molecules. Med Res Rev. (2019) 39:265–301. 10.1002/med.21530 30215856

[48] Sharma P Allison JP. The future of immune checkpoint therapy. Science. (2015) 348:56–61. 10.1126/science.aaa8172 25838373

[49] Sharma P Allison JP. Immune checkpoint targeting in cancer therapy: toward combination strategies with curative potential. Cell. (2015) 161:205–14. 10.1016/j.cell.2015.03.030 25860605 PMC5905674

[50] Sakuishi K Apetoh L Sullivan JM Blazar BR Kuchroo VK Anderson AC. Targeting Tim-3 and PD-1 pathways to reverse T cell exhaustion and restore anti-tumor immunity. J Exp Med. (2010) 207:2187–94. 10.1084/jem.20100643 20819927 PMC2947065

[51] Long L Zhang X Chen F Pan Q Phiphatwatchara P Zeng Y. The promising immune checkpoint LAG-3: from tumor microenvironment to cancer immunotherapy. Genes Cancer. (2018) 9:176–89. 10.18632/genesandcancer.180 30603054 PMC6305110

[52] Brown NF Carter TJ Ottaviani D Mulholland P. Harnessing the immune system in glioblastoma. Br J Cancer. (2018) 119:1171. 10.1038/s41416-018-0258-8 30393372 PMC6251037

[53] Chen DS Mellman I. Elements of cancer immunity and the cancer-immune set point. Nature. (2017) 541:321–30. 10.1038/nature21349 28102259

[54] Zaretsky JM Garcia-Diaz A Shin DS Escuin-Ordinas H Hugo W Hu-Lieskovan S. Mutations associated with acquired resistance to PD-1 blockade in melanoma. N Engl J Med. (2016) 375:819–29. 10.1056/NEJMoa1604958 27433843 PMC5007206

[55] Gao J Shi LZ Zhao H Chen J Xiong L He Q. Loss of IFN-γ pathway genes in tumor cells as a mechanism of resistance to Anti-CTLA-4 therapy. Cell. (2016) 167:397–404 e9. 10.1016/j.cell.2016.08.069 27667683 PMC5088716

[56] Chowell D Morris LGT Grigg CM Weber JK Samstein RM Makarov V. Patient HLA class I genotype influences cancer response to checkpoint blockade immunotherapy. Science. (2018) 359:582–7. 10.1126/science.aao4572 29217585 PMC6057471

[57] Murakami N Motwani S Riella LV. Renal complications of immune checkpoint blockade. Curr Problems Cancer. (2017) 41:100–10. 10.1016/j.currproblcancer.2016.12.004 28189263 PMC5440194

[58] Delaunay M Caron P Sibaud V Godillot C Collot S Milia J. [Toxicity of immune checkpoints inhibitors]. Revue Mal Respir. (2018) 35:1028–38. 10.1016/j.rmr.2017.08.006 30213624

[59] Sharma P. Immune checkpoint therapy and the search for predictive biomarkers. Cancer J. (2016) 22:68–72. 10.1097/PPO.0000000000000185 27111900 PMC4847150

[60] Joyce JA Fearon DT. T cell exclusion, immune privilege, and the tumor microenvironment. Science. (2015) 348:74–80. 10.1126/science.aaa6204 25838376

[61] Fritz V Fajas L. Metabolism and proliferation share common regulatory pathways in cancer cells. Oncogene. (2010) 29:4369–77. 10.1038/onc.2010.182 20514019 PMC3004916

[62] Bell JC Stojdl DF Lichty B Knowles S Marius R Atkins H. Exploiting tumor-specific defects in the interferon pathway with a previously unknown oncolytic virus. Nat Med. (2000) 6:821–5. 10.1038/77558 10888934

[63] Rabinovich GA Gabrilovich D Sotomayor EM. Immunosuppressive strategies that are mediated by tumor cells. Ann Rev Immunol. (2007) 25:267–96. 10.1146/annurev.immunol.25.022106.141609 17134371 PMC2895922

[64] Ilkow CS Marguerie M Batenchuk C Mayer J Ben Neriah D Cousineau S. Reciprocal cellular cross-talk within the tumor microenvironment promotes oncolytic virus activity. Nat Med. (2015) 21:530–6. 10.1038/nm.3848 25894825

[65] Mansour M Palese P Zamarin D. Oncolytic specificity of newcastle disease virus is mediated by selectivity for apoptosis-resistant cells. J Virol. (2011) 85:6015–23. 10.1128/JVI.01537-10 21471241 PMC3126310

[66] Strong JE Coffey MC Tang D Sabinin P Lee PWK. The molecular basis of viral oncolysis: usurpation of the Ras signaling pathway by reovirus. EMBO J. (1998) 17:3351–62. 10.1093/emboj/17.12.3351 9628872 PMC1170673

[67] Chiocca EA Rabkin SD. Oncolytic viruses and their application to cancer immunotherapy. Cancer Immunol Res. (2014) 2:295–300. 10.1158/2326-6066.CIR-14-0015 24764576 PMC4303349

[68] Conry RM Westbrook B McKee S Norwood TG. Talimogene laherparepvec: first in class oncolytic virotherapy. Hum Vaccin Immunother. (2018) 14:839–46. 10.1080/21645515.2017.1412896 29420123 PMC5893211

[69] Brown MC Holl EK Boczkowski D Dobrikova E Mosaheb M Chandramohan V. Cancer immunotherapy with recombinant poliovirus induces IFN-dominant activation of dendritic cells and tumor antigen-specific CTLs. Sci Transl Med. (2017) 9:eaan4220. 10.1126/scitranslmed.aan4220 28931654 PMC6034685

[70] Desjardins A Gromeier M Herndon JE II Beaubier N Bolognesi DP Friedman AH. Recurrent glioblastoma treated with recombinant poliovirus. N Engl J Med. (2018) 379:150–61. 10.1056/NEJMoa1716435 29943666 PMC6065102

[71] Eissa IR Bustos-Villalobos I Ichinose T Matsumura S Naoe Y Miyajima N. The current status and future prospects of oncolytic viruses in clinical trials against melanoma, glioma, pancreatic, and breast cancers. Cancers. (2018) 10:E356. 10.3390/cancers10100356 30261620 PMC6210336

[72] Abe T Marutani Y Shoji I. Cytosolic DNA-sensing immune response and viral infection. Microbiol Immunol. (2019) 63:51–64. 10.1111/1348-0421.12669 30677166 PMC7168513

[73] Chow KT Gale M Loo YM. RIG-I and other RNA sensors in antiviral immunity. Ann Rev Immunol. (2018) 36:667–94. 10.1146/annurev-immunol-042617-053309 29677479

[74] Said EA Tremblay N Al-Balushi MS Al-Jabri AA Lamarre D. Viruses seen by our cells: the role of viral RNA sensors. J Immunol Res. (2018) 2018:9480497. 10.1155/2018/9480497 29854853 PMC5952511

[75] Stanford MM Breitbach CJ Bell JC McFadden G Innate immunity, tumor microenvironment and oncolytic virus therapy: friends or foes? Curr Opin Molecul Therapeut. (2008) 10:32–7. 18228179

[76] Zitvogel L Galluzzi L Kepp O Smyth MJ Kroemer G. Type I interferons in anticancer immunity. Nat Rev Immunol. (2015) 15:405–14. 10.1038/nri3845 26027717

[77] Corrales L McWhirter SM Dubensky TW Gajewski TF. The host STING pathway at the interface of cancer and immunity. J Clin Invest. (2016) 126:2404–11. 10.1172/JCI86892 27367184 PMC4922692

[78] Khoo LT Chen LY. Role of the cGAS-STING pathway in cancer development and oncotherapeutic approaches. EMBO Rep. (2018) 19:e46935. 10.15252/embr.201846935 30446584 PMC6280650

[79] Sadler AJ Williams BRG. Interferon-inducible antiviral effectors. Nat Rev Immunol. (2008) 8:559–68. 10.1038/nri2314 18575461 PMC2522268

[80] Hobeika AC Subramaniam PS Johnson HM. IFNα induces the expression of the cyclin-dependent kinase inhibitor p21 in human prostate cancer cells. Oncogene. (1997) 14:1165–70. 10.1038/sj.onc.1200939 9121765

[81] Maeda S Wada H Naito Y Nagano H Simmons S Kagawa Y. Interferon-α acts on the S/G2/M phases to induce apoptosis in the G1 phase of an IFNAR2-expressing hepatocellular carcinoma cell line. J Biol Chem. (2014) 289:23786–95. 10.1074/jbc.M114.551879 25012666 PMC4156060

[82] Nguyen KB Salazar-Mather TP Dalod MY Van Deusen JB Wei XQ Liew FY. Coordinated and distinct roles for IFN-, IL-12, and IL-15 regulation of NK cell responses to viral infection. J Immunol. (2002) 169:4279–87. 10.4049/jimmunol.169.8.4279 12370359

[83] Gidlund M Örn A Wigzell H Senik A Gresser I. Enhanced NK cell activity in mice injected with interferon and interferon inducers. Nature. (1978) 273:759–61. 10.1038/273759a0 566386

[84] Castro F Cardoso AP Gonçalves RM Serre K Oliveira MJ Interferon-gamma at the crossroads of tumor immune surveillance or evasion. Front Immunol. (2018) 9:847 10.3389/fimmu.2018.00847 29780381 PMC5945880

[85] Montoya M Schiavoni G Mattel F Gresser I Belardelli F Borrow P. Type I interferons produced by dendritic cells promote their phenotypic and functional activation. Blood. (2002) 99:3263–71. 10.1182/blood.V99.9.3263 11964292

[86] Curtsinger JM Valenzuela JO Agarwal P Lins D Mescher MF Cutting edge: type I IFNs provide a third signal to CD8 T cells to stimulate clonal expansion and differentiation. J Immunol. (2005) 174:4465–9. 10.4049/jimmunol.174.8.4465 15814665

[87] Marrack P Kappler J Mitchell T. Type I interferons keep activated T cells alive. J Exp Med. (1999) 189:521–9. 10.1084/jem.189.3.521 9927514 PMC2192920

[88] Le Bon A Tough DF. Type I interferon as a stimulus for cross-priming. Cytokine Growth Factor Rev. (2008) 19:33–40. 10.1016/j.cytogfr.2007.10.007 18068417

[89] Müller L Aigner P Stoiber D. Type I interferons and natural killer cell regulation in cancer. Front Immunol. (2017) 8:304. 10.3389/fimmu.2017.00304 28408907 PMC5374157

[90] Brinkmann V Geiger T Alkan S Heusser CH Interferon α increases the frequency of interferon y-producing human CD4 + T cells. J Exp Med. (1993) 178:1655–63. 10.1084/jem.178.5.1655 8228812 PMC2191249

[91] Allagui F Achard C Panterne C Combredet C Labarrière N Dréno B. Modulation of the Type I interferon response defines the sensitivity of human melanoma cells to oncolytic measles virus. Curr Gene Ther. (2017) 16:419–28. 10.2174/1566523217666170102110502 28042780

[92] Paglino JC Andres W van den Pol AN Autonomous parvoviruses neither stimulate nor are inhibited by the type I interferon response in human normal or cancer cells. J Virol. (2014) 88:4932–42. 10.1128/JVI.03508-13 24554651 PMC3993814

[93] Qian S Fan W Liu T Wu M Zhang H Cui X. Seneca valley virus suppresses host Type I interferon production by targeting adaptor proteins MAVS, TRIF, and TANK for Cleavage. J Virol. (2017) 91:e00823–17. 10.1128/JVI.00823-17 28566380 PMC5533933

[94] Workenhe ST Mossman KL. Oncolytic virotherapy and immunogenic cancer cell death: sharpening the sword for improved cancer treatment strategies. Mol Ther. (2014) 22:251–6. 10.1038/mt.2013.220 24048442 PMC3916036

[95] Redza-Dutordoir M Averill-Bates DA. Activation of apoptosis signalling pathways by reactive oxygen species. Biochim Biophys Acta. (2016) 1863:2977–92. 10.1016/j.bbamcr.2016.09.012 27646922

[96] Elliott MR Chekeni FB Trampont PC Lazarowski ER Kadl A Walk SF. Nucleotides released by apoptotic cells act as a find-me signal. Nature. (2010) 461:282–6. 10.1038/nature08296 19741708 PMC2851546

[97] Ibrahim ZA Armour CL Phipps S Sukkar MB RAGE and TLRs: relatives, friends or neighbours? Mol Immunol. (2013) 56:739–44. 10.1016/j.molimm.2013.07.008 23954397

[98] Gardai SJ McPhillips KA Frasch SC Janssen WJ Starefeldt A Murphy-Ullrich JE. Cell-surface calreticulin initiates clearance of viable or apoptotic cells through trans-activation of LRP on the phagocyte. Cell. (2005) 123:321–34. 10.1016/j.cell.2005.08.032 16239148

[99] Galluzzi L Vitale I Aaronson SA Abrams JM Adam D Agostinis P. Molecular mechanisms of cell death: recommendations of the Nomenclature Committee on Cell Death 2018. Cell Death Differ. (2018) 25:486–541. 10.1038/s41418-018-0102-y 29362479 PMC5864239

[100] Breitbach CJ Burke J Jonker D Stephenson J Haas AR Chow LQ. Intravenous delivery of a multi-mechanistic cancer-targeted oncolytic poxvirus in humans. Nature. (2011) 477:99–102. 10.1038/nature10358 21886163

[101] Breitbach CJ Arulanandam R De Silva N Thorne SH Patt R Daneshmand M. Oncolytic vaccinia virus disrupts tumor-associated vasculature in humans. Cancer Res. (2013) 73:1265–75. 10.1158/0008-5472.CAN-12-2687 23393196

[102] Arulanandam R Batenchuk C Angarita FA Ottolino-Perry K Cousineau S Mottashed A. VEGF-mediated induction of PRD1-BF1/Blimp1 expression sensitizes tumor vasculature to oncolytic virus infection. Cancer Cell. (2015) 28:210–24. 10.1016/j.ccell.2015.06.009 26212250

[103] Benencia F Courreges MC Conejo-Garcia JR Buckanovich RJ Zhang L Carroll RH. Oncolytic HSV exerts direct antiangiogenic activity in ovarian carcinoma. Hum Gene Ther. (2005) 16:765–78. 10.1089/hum.2005.16.765 15960607

[104] Cinatl J Jr Michaelis M Driever PH Cinatl J Hrabeta J Suhan T. Multimutated herpes simplex virus g207 is a potent inhibitor of angiogenesis. Neoplasia. (2004) 6:725–35. 10.1593/neo.04265 15720798 PMC1531676

[105] Marchini A Scott EM Rommelaere J. Overcoming barriers in oncolytic virotherapy with HDAC inhibitors and immune checkpoint blockade. Viruses. (2016) 8:9. 10.3390/v8010009 26751469 PMC4728569

[106] Kostense S Koudstaal W Sprangers M Weverling GJ Penders G Helmus N. Adenovirus types 5 and 35 seroprevalence in AIDS risk groups supports type 35 as a vaccine vector. AIDS. (2004) 18:1213–16. 10.1097/00002030-200405210-00019 15166541

[107] Whitley RJ Herpesviruses In: Baron S, editor. Medical Microbiology. Galveston, TX: University of Texas Medical Branch at Galveston (1996). 21413307

[108] Adair RA Roulstone V Scott KJ Morgan R Nuovo GJ Fuller M. Cell carriage, delivery, and selective replication of an oncolytic virus in tumor in patients. Sci Transl Med. (2012) 4:138ra77. 10.1126/scitranslmed.3003578 22700953 PMC3893925

[109] Ilett E Kottke T Donnelly O Thompson J Willmon C Diaz R. Cytokine conditioning enhances systemic delivery and therapy of an oncolytic virus. Mol Ther. (2014) 22:1851–63. 10.1038/mt.2014.118 24957982 PMC4428400

[110] Russell SJ Federspiel MJ Peng KW Tong C Dingli D Morice WG. Remission of disseminated cancer after systemic oncolytic virotherapy. Mayo Clin Proc. (2014) 89:926–33. 10.1016/j.mayocp.2014.04.003 24835528 PMC4225126

[111] Hagedorn C Kreppel F. Capsid engineering of adenovirus vectors: overcoming early vector-host interactions for therapy. Hum Gene Ther. (2017) 28:820–32. 10.1089/hum.2017.139 28854810

[112] Wonganan P Croyle MA. PEGylated adenoviruses: from mice to monkeys. Viruses. (2010) 2:468–502. 10.3390/v2020468 21994645 PMC3185605

[113] Kim Y Clements DR Sterea AM Jang HW Gujar SA Lee PW. Dendritic cells in oncolytic virus-based anti-cancer therapy. Viruses. (2015) 7:6506–25. 10.3390/v7122953 26690204 PMC4690876

[114] de Graaf JF de Vor L Fouchier RAM van den Hoogen BG. Armed oncolytic viruses: a kick-start for anti-tumor immunity. Cytokine Growth Factor Rev. (2018) 41:28–39. 10.1016/j.cytogfr.2018.03.006 29576283 PMC7108398

[115] Senzer NN Kaufman HL Amatruda T Nemunaitis M Reid T Daniels G. Phase II clinical trial of a granulocyte-macrophage colony-stimulating factor-encoding, second-generation oncolytic herpesvirus in patients with unresectable metastatic melanoma. J Clin Oncol. (2009) 27:5763–71. 10.1200/JCO.2009.24.3675 19884534

[116] Andtbacka RH Kaufman HL Collichio F Amatruda T Senzer N Chesney J. Talimogene laherparepvec improves durable response rate in patients with advanced melanoma. J Clin Oncol. (2015) 33:2780–8. 10.1200/JCO.2014.58.3377 26014293

[117] Liu BL Robinson M Han ZQ Branston RH English C Reay P. ICP34.5 deleted herpes simplex virus with enhanced oncolytic, immune stimulating, and anti-tumour properties. Gene Ther. (2003) 10:292–303. 10.1038/sj.gt.3301885 12595888

[118] Fajardo CA Guedan S Rojas LA Moreno R Arias-Badia M de Sostoa J. Oncolytic adenoviral delivery of an EGFR-targeting t-cell engager improves antitumor efficacy. Cancer Res. (2017) 77:2052–63. 10.1158/0008-5472.CAN-16-1708 28143835

[119] Freedman JD Duffy MR Lei-Rossmann J Muntzer A Scott EM Hagel J. An oncolytic virus expressing a t-cell engager simultaneously targets cancer and immunosuppressive stromal cells. Cancer Res. (2018) 78:6852–65. 10.1158/0008-5472.CAN-18-1750 30449733

[120] Bartee MY Dunlap KM Bartee E. Tumor-localized secretion of soluble PD1 enhances oncolytic virotherapy. Cancer Res. (2017) 77:2952–63. 10.1158/0008-5472.CAN-16-1638 28314785 PMC5457316

[121] Dias JD Hemminki O Diaconu I Hirvinen M Bonetti A Guse K. Targeted cancer immunotherapy with oncolytic adenovirus coding for a fully human monoclonal antibody specific for CTLA-4. Gene Ther. (2012) 19:988–98. 10.1038/gt.2011.176 22071969

[122] Engeland CE Grossardt C Veinalde R Bossow S Lutz D Kaufmann JK. CTLA-4 and PD-L1 checkpoint blockade enhances oncolytic measles virus therapy. Mol Ther. (2014) 22:1949–59. 10.1038/mt.2014.160 25156126 PMC4429737

[123] Kleinpeter P Fend L Thioudellet C Geist M Sfrontato N Koerper V. Vectorization in an oncolytic vaccinia virus of an antibody, a Fab and a scFv against programmed cell death−1 (PD-1) allows their intratumoral delivery and an improved tumor-growth inhibition. Oncoimmunology. (2016) 5:e1220467. 10.1080/2162402X.2016.1220467 27853644 PMC5087307

[124] Kvistborg P Philips D Kelderman S Hageman L Ottensmeier C Joseph-Pietras D. Anti-CTLA-4 therapy broadens the melanoma-reactive CD8+ T cell response. Sci Transl Med. (2014) 6:254ra128. 10.1126/scitranslmed.3008918 25232180

[125] Simpson TR Li F Montalvo-Ortiz W Sepulveda MA Bergerhoff K Arce F. Fc-dependent depletion of tumor-infiltrating regulatory T cells co-defines the efficacy of anti-CTLA-4 therapy against melanoma. J Exp Med. (2013) 210:1695–710. 10.1084/jem.20130579 23897981 PMC3754863

[126] Tysome JR Li X Wang S Wang P Gao D Du P. A novel therapeutic regimen to eradicate established solid tumors with an effective induction of tumor-specific immunity. Clin Cancer Res. (2012) 18:6679–89. 10.1158/1078-0432.CCR-12-0979 23091113

[127] Aitken AS Roy DG Bourgeois-Daigneault MC. Taking a stab at cancer; oncolytic virus-mediated anti-cancer vaccination strategies. Biomedicines. (2017) 5:E3. 10.3390/biomedicines5010003 28536346 PMC5423491

[128] Pol JG Atherton MJ Bridle BW Stephenson KB Le Boeuf F Hummel JL. Development and applications of oncolytic Maraba virus vaccines. Oncolytic Virother. (2018) 7:117–28. 10.2147/OV.S154494 30538968 PMC6263248

[129] Ilett E Kottke T Thompson J Rajani K Zaidi S Evgin L. Prime-boost using separate oncolytic viruses in combination with checkpoint blockade improves anti-tumour therapy. Gene Ther. (2017) 24:21–30. 10.1038/gt.2016.70 27779616 PMC5387692

[130] Ottolino-Perry K Diallo JS Lichty BD Bell JC McCart JA. Intelligent design: combination therapy with oncolytic viruses. Mol Ther. (2010) 18:251–63. 10.1038/mt.2009.283 20029399 PMC2839289

[131] Wennier ST Liu J McFadden G. Bugs and drugs: oncolytic virotherapy in combination with chemotherapy. Curr Pharm Biotechnol. (2012) 13:1817–33. 10.2174/138920112800958850 21740354 PMC4373447

[132] Guedan S Alemany R. CAR-T cells and oncolytic viruses: joining forces to overcome the solid tumor challenge. Front Immunol. (2018) 9:2460. 10.3389/fimmu.2018.02460 30405639 PMC6207052

[133] LaRocca CJ Warner SG. Oncolytic viruses and checkpoint inhibitors: combination therapy in clinical trials. Clin Transl Med. (2018) 7:35. 10.1186/s40169-018-0214-5 30426287 PMC6234197

[134] Zamarin D Holmgaard RB Subudhi SK Park JS Mansour M Palese P. Localized oncolytic virotherapy overcomes systemic tumor resistance to immune checkpoint blockade immunotherapy. Sci Transl Med. (2014) 6:226ra32. 10.1126/scitranslmed.3008095 24598590 PMC4106918

[135] Durham NM Mulgrew K McGlinchey K Monks NR Ji H Herbst R. Oncolytic VSV primes differential responses to immuno-oncology therapy. Mol Ther. (2017) 25:1917–32. 10.1016/j.ymthe.2017.05.006 28578991 PMC5542805

[136] Liu Z Ravindranathan R Kalinski P Guo ZS Bartlett DL. Rational combination of oncolytic vaccinia virus and PD-L1 blockade works synergistically to enhance therapeutic efficacy. Nat Commun. (2017) 8:14754. 10.1038/ncomms14754 28345650 PMC5378974

[137] Woller N Gurlevik E Fleischmann-Mundt B Schumacher A Knocke S Kloos AM. Viral Infection of Tumors Overcomes Resistance to PD-1-immunotherapy by Broadening Neoantigenome-directed T-cell Responses. Mol Ther. (2015) 23:1630–40. 10.1038/mt.2015.115 26112079 PMC4817928

[138] Samson A Scott KJ Taggart D West EJ Wilson E Nuovo GJ. Intravenous delivery of oncolytic reovirus to brain tumor patients immunologically primes for subsequent checkpoint blockade. Sci Transl Med. (2018) 10:eaam7577. 10.1126/scitranslmed.aam7577 29298869 PMC6276984

[139] Bourgeois-Daigneault MC Roy DG Aitken AS El Sayes N Martin NT Varette O. Neoadjuvant oncolytic virotherapy before surgery sensitizes triple-negative breast cancer to immune checkpoint therapy. Sci Transl Med. (2018) 10:eaao1641. 10.1126/scitranslmed.aao1641 29298865

[140] Garcia-Diaz A Shin DS Moreno BH Saco J Escuin-Ordinas H Rodriguez GA. Interferon receptor signaling pathways regulating PD-L1 and PD-L2 expression. Cell Rep. (2017) 19:1189–201. 10.1016/j.celrep.2017.04.031 28494868 PMC6420824

[141] Chesney J Puzanov I Collichio F Singh P Milhem MM Glaspy J. Randomized, open-label phase II study evaluating the efficacy and safety of talimogene laherparepvec in combination with ipilimumab versus ipilimumab alone in patients with advanced, unresectable melanoma. J Clin Oncol. (2018) 36:1658–67. 10.1200/JCO.2017.73.7379 28981385 PMC6075852

[142] Ribas A Dummer R Puzanov I VanderWalde A Andtbacka RHI Michielin O. Oncolytic virotherapy promotes intratumoral T cell infiltration and improves anti-PD-1 immunotherapy. Cell. (2017) 170:1109–19.e10. 10.1016/j.cell.2017.08.027 28886381 PMC8034392

[143] Marchini A Bonifati S Scott EM Angelova AL Rommelaere J. Oncolytic parvoviruses: from basic virology to clinical applications. Virol J. (2015) 12:6. 10.1186/s12985-014-0223-y 25630937 PMC4323056

[144] Bretscher C Marchini A. H-1 parvovirus as a cancer-killing agent: past, present, and future. Viruses. (2019) 11:E562. 10.3390/v11060562 31216641 PMC6630270

[145] Nuesch JP Lacroix J Marchini A Rommelaere J. Molecular pathways: rodent parvoviruses–mechanisms of oncolysis and prospects for clinical cancer treatment. Clin Cancer Res. (2012) 18:3516–23. 10.1158/1078-0432.CCR-11-2325 22566376

[146] Angelova AL Geletneky K Nüesch JPF Rommelaere J. Tumor selectivity of oncolytic parvoviruses: from in vitro and animal models to cancer patients. Front Bioeng Biotechnol. (2015) 3:55. 10.3389/fbioe.2015.00055 25954743 PMC4406089

[147] Grekova S Aprahamian M Giese N Schmitt S Giese T Falk CS. Immune cells participate in the oncosuppressive activity of parvovirus H-1PV and are activated as a result of their abortive infection with this agent. Cancer Biol Ther. (2010) 10:1280–9. 10.4161/cbt.10.12.13455 21124075

[148] Mattei LM Cotmore SF Tattersall P Iwasaki A. Parvovirus evades interferon-dependent viral control in primary mouse embryonic fibroblasts. Virology. (2013) 442:20–7. 10.1016/j.virol.2013.03.020 23676303 PMC3767977

[149] Schlehofer JR Rentrop M Mannel DN Parvoviruses are inefficient in inducing interferon-β, tumor necrosis factor-α, or interleukin-6 in mammalian cells. Med Microbiol Immunol. (1992) 181:153–64. 10.1007/BF00202055 1522825

[150] Angelova A Rommelaere J Immune adjuvant capacities of oncolytic rodent protoparvoviruses. Viruses. (2019) 11:415 10.3390/v11050415 PMC6563271 31060205

[151] Geletneky K Nuesch JP Angelova A Kiprianova I Rommelaere J. Double-faceted mechanism of parvoviral oncosuppression. Curr Opin Virol. (2015) 13:17–24. 10.1016/j.coviro.2015.03.008 25841215

[152] Hristov G Krämer M Li J El-Andaloussi N Mora R Daeffler L. Through its nonstructural protein NS1, parvovirus H-1 induces apoptosis via accumulation of reactive oxygen species. J Virol. (2010) 84:5909–22. 10.1128/JVI.01797-09 20375165 PMC2876649

[153] Di Piazza M Mader C Geletneky K Herrero Calle M Weber E Schlehofer J. Cytosolic activation of cathepsins mediates parvovirus H-1-induced killing of cisplatin and TRAIL-resistant glioma cells. J Virol. (2007) 81:4186–98. 10.1128/JVI.02601-06 17287256 PMC1866092

[154] Mészáros I Tóth R Olasz F Tijssen P Zádori Z. The SAT protein of porcine parvovirus accelerates viral spreading through induction of irreversible endoplasmic reticulum stress. J Virol. (2017) 91:e00627–17. 10.1128/JVI.00627-17 28566374 PMC5533901

[155] Moehler M Zeidler M Schede J Rommelaere J Galle PR Cornelis JJ Oncolytic parvovirus H1 induces release of heat-shock protein HSP72 in susceptible human tumor cells but may not affect primary immune cells. Cancer Gene Ther. (2003) 10:477–80. 10.1038/sj.cgt.7700591 12768193

[156] Raykov Z Grekova SP Hörlein R Leuchs B Giese T Giese NA. TLR-9 Contributes to the antiviral innate immune sensing of rodent parvoviruses MVMp and H-1PV by normal human immune cells. PLoS ONE. (2013) 8:e55086. 10.1371/journal.pone.0055086 23383065 PMC3558501

[157] Geletneky K Leoni AL Pohlmeyer-Esch G Loebhard S Baetz A Leuchs B. Pathology, organ distribution, and immune response after single and repeated intravenous injection of rats with clinical-grade parvovirus H1. Compar Med. (2015) 65:23–35. 25730754 PMC4396926

[158] Moralès O Richard A Martin N Mrizak D Sénéchal M Miroux C Activation of a helper and not regulatory human CD4+ T cell response by oncolytic H-1 parvovirus. PLoS ONE. (2012) 7:e32197 10.1371/journal.pone.0032197 22359669 PMC3281136

[159] Bhat R Dempe S Dinsart C Rommelaere J. Enhancement of NK cell antitumor responses using an oncolytic parvovirus. Int J Cancer. (2011) 128:908–19. 10.1002/ijc.25415 20473905

[160] Angelova AL Grekova SP Heller A Kuhlmann O Soyka E Giese T. Complementary induction of immunogenic cell death by oncolytic parvovirus H-1PV and gemcitabine in pancreatic cancer. J Virol. (2014) 88:5263–76. 10.1128/JVI.03688-13 24574398 PMC4019131

[161] Asea A. Initiation of the immune response by extracellular Hsp72: chaperokine activity of Hsp72. Curr. Immunol. Rev. (2006) 2:209–15. 17502920 10.2174/157339506778018514 PMC1868403

[162] Moehler M Sieben M Roth S Springsguth F Leuchs B Zeidler M. Activation of the human immune system by chemotherapeutic or targeted agents combined with the oncolytic parvovirus H-1. BMC Cancer. (2011) 11:464. 10.1186/1471-2407-11-464 22029859 PMC3234202

[163] Sieben M Schäfer P Dinsart C Galle PR Moehler M. Activation of the human immune system via toll-like receptors by the oncolytic parvovirus H-1. Int J Cancer. (2013) 132:2548–56. 10.1002/ijc.27938 23151948

[164] Moehler MH Zeidler M Wilsberg V Cornelis JJ Woelfel T Rommelaere J. Parvovirus H-1-induced tumor cell death enhances human immune response *in vitro* via increased phagocytosis, maturation, and cross-presentation by dendritic cells. Human Gene Therapy. (2005) 16:996–1005. 10.1089/hum.2005.16.996 16076257

[165] Raykov Z Grekova S Galabov AS Balboni G Koch U Aprahamian M. Combined oncolytic and vaccination activities of parvovirus H-1 in a metastatic tumor model. Oncol Rep. (2007) 17:1493–9. 10.3892/or.17.6.1493 17487410

[166] Geletneky K Kiprianova I Ayache A Koch R Herrero M Calle Y. Regression of advanced rat and human gliomas by local or systemic treatment with oncolytic parvovirus H-1 in rat models. Neuro-Oncology. (2010) 12:804–14. 10.1093/neuonc/noq023 20299703 PMC2940670

[167] Grekova S Zawatzky R Horlein R Cziepluch C Mincberg M Davis C Activation of an antiviral response in normal but not transformed mouse cells: a new determinant of minute virus of mice oncotropism. J Virol. (2010) 84:516–31. 10.1128/JVI.01618-09 19864388 PMC2798439

[168] Grekova SP Aprahamian M Daeffler L Leuchs B Angelova A Giese T. Interferon γ improves the vaccination potential of oncolytic parvovirus H-1PV for the treatment of peritoneal carcinomatosis in pancreatic cancer. Cancer Biol Ther. (2011) 12:888–95. 10.4161/cbt.12.10.17678 22024742 PMC3280904

[169] Rommelaere J Geletneky K Angelova AL Daeffler L Dinsart C Kiprianova I. Oncolytic parvoviruses as cancer therapeutics. Cytokine Growth Factor Rev. (2010) 21:185–95. 10.1016/j.cytogfr.2010.02.011 20211577

[170] Quail DF Joyce JA. The microenvironmental landscape of brain tumors. Cancer Cell. (2017) 31:326–341. 10.1016/j.ccell.2017.02.009 28292436 PMC5424263

[171] Alexandrov LB Nik-Zainal S Wedge DC Aparicio SA Behjati S Biankin AV. Signatures of mutational processes in human cancer. Nature. (2013) 500:415–21. 10.1038/nature12477 23945592 PMC3776390

[172] Bagley SJ Desai AS Linette GP June CH O'Rourke DM. CAR T-cell therapy for glioblastoma: recent clinical advances and future challenges. Neuro Oncol. (2018) 20:1429–38. 10.1093/neuonc/noy032 29509936 PMC6176794

[173] Geletneky K Kiprianova I Ayache A Koch R Herrero YCM Deleu L. Regression of advanced rat and human gliomas by local or systemic treatment with oncolytic parvovirus H-1 in rat models. Neuro Oncol. (2010) 12:804–14. 20299703 10.1093/neuonc/noq023 PMC2940670

[174] Geletneky K Huesing J Rommelaere J Schlehofer JR Leuchs B Dahm M. Phase I/IIa study of intratumoral/intracerebral or intravenous/intracerebral administration of Parvovirus H-1 (ParvOryx) in patients with progressive primary or recurrent glioblastoma multiforme: ParvOryx01 protocol. BMC Cancer. (2012) 12:99. 10.1186/1471-2407-12-99 22436661 PMC3425127

[175] Ungerechts G Engeland CE Buchholz CJ Eberle J Fechner H Geletneky K. Virotherapy research in germany: from engineering to translation. Hum Gene Ther. (2017) 28:800–19. 10.1089/hum.2017.138 28870120

[176] Geletneky K Hajda J Angelova AL Leuchs B Capper D Bartsch AJ. Oncolytic H-1 parvovirus shows safety and signs of immunogenic activity in a first phase I/IIa glioblastoma trial. Mol Ther. (2017) 25:2620–34. 10.1016/j.ymthe.2017.08.016 28967558 PMC5768665

[177] Hajda J Lehmann M Krebs O Kieser M Geletneky K Jager D. A non-controlled, single arm, open label, phase II study of intravenous and intratumoral administration of ParvOryx in patients with metastatic, inoperable pancreatic cancer: ParvOryx02 protocol. BMC Cancer. (2017) 17:576. 10.1186/s12885-017-3604-y 28851316 PMC5574242

[178] Angelova AL Barf M Geletneky K Unterberg A Rommelaere J. Immunotherapeutic potential of oncolytic H-1 parvovirus: hints of glioblastoma microenvironment conversion towards immunogenicity. Viruses. (2017) 9:E382. 10.3390/v9120382 29244745 PMC5744156

[179] Angelova AL Aprahamian M Grekova SP Hajri A Leuchs B Giese NA. Improvement of gemcitabine-based therapy of pancreatic carcinoma by means of oncolytic parvovirus H-1PV. Clin Cancer Res. (2009) 15:511–9. 10.1158/1078-0432.CCR-08-1088 19147756

[180] Li J Bonifati S Hristov G Marttila T Valmary-Degano S Stanzel S. Synergistic combination of valproic acid and oncolytic parvovirus H-1PV as a potential therapy against cervical and pancreatic carcinomas. EMBO Mol Med. (2013) 5:1537–55. 10.1002/emmm.201302796 24092664 PMC3799578

[181] Heinrich B Goepfert K Delic M Galle PR Moehler M.Influence of the oncolytic parvovirus H-1, CTLA-4 antibody tremelimumab and cytostatic drugs on the human immune system in a human *in vitro* model of colorectal cancer cells. Onco Targets Ther. (2013) 6:1119–27. 10.2147/OTT.S49371 23986643 PMC3754820

[182] Geletneky K Angelova A Leuchs B Bartsch A Capper D Hajda J ATNT-07. Favorable response of patients with glioblastoma at second or third recurrence to repeated injection of oncolytic parvovirus H-1 in combination with Bevacicumab. Neuro Oncol. (2015) 17(Suppl 5): v11 10.1093/neuonc/nov205.07

[183] Geletneky K Bartsch A Weiss C Bernhard H Marchini A Rommelaere J ATIM-40. High rate of objective anti.tumor response in 9 patients with glioblastoma after viro-immunotherapy with oncolytic parvovirus H-1 in combination with bavacicumab and PD-1 checkpoint blockade. Neuro-Oncology. (2018) 20:vi10 10.1093/neuonc/noy148.035

[184] Karsten G Weiss C Bernhard H Capper D Leuchs B Marchini A ATIM-29. First clinical observation of improved anti-tumor effects of viro-immunotherapy with oncolytic parvovirus H-1 in combination with PD-1 checkpoint blockade and bevacicumab in patients with recurrent glioblastoma. Neuro-Oncology. (2016) 18:Pages vi24 10.1093/neuonc/now212.094

[185] Grekova SP Aprahamian M Giese NA Bour G Giese T Grewenig A Genomic CpG enrichment of oncolytic parvoviruses as a potent anticancer vaccination strategy for the treatment of pancreatic adenocarcinoma. J Vaccines Vaccin. (2014) 5:2 10.4172/2157-7560.1000227

[186] Lavie M Struyf S Stroh-Dege A Rommelaere J Van Damme J Dinsart C. Capacity of wild-type and chemokine-armed parvovirus H-1PV for inhibiting neo-angiogenesis. Virology. (2013) 447:221–32. 10.1016/j.virol.2013.09.019 24210118

[187] El-Andaloussi N Endele M Leuchs B Bonifati S Kleinschmidt J Rommelaere J. Novel adenovirus-based helper system to support production of recombinant parvovirus. Cancer Gene Ther. (2011) 18:240–9. 10.1038/cgt.2010.73 21102423

[188] El-Andaloussi N Barbara L Serena B Rommelaere J Marchini A Efficient recombinant parvovirus production with the help of adenovirus- derived systems. J Vis Exp. (2011) 2012:e3518 10.3791/3518 PMC3468171 22546707

[189] El-Andaloussi N Bonifati S Kaufmann JK Mailly L Daeffler L Deryckere F. Generation of an adenovirus-parvovirus chimera with enhanced oncolytic potential. J Virol. (2012) 86:10418–31. 10.1128/JVI.00848-12 22787235 PMC3457320

